# Linking Taxonomic, Phylogenetic and Functional Plant Diversity with Ecosystem Services of Cliffs and Screes in Greece

**DOI:** 10.3390/plants10050992

**Published:** 2021-05-17

**Authors:** Maria Panitsa, Ioannis P. Kokkoris, Konstantinos Kougioumoutzis, Anna Kontopanou, Ioannis Bazos, Arne Strid, Panayotis Dimopoulos

**Affiliations:** 1 Laboratory of Botany, Department of Biology, Division of Plant Biology, University of Patras, 26504 Patras, Greece; ipkokkoris@upatras.gr (I.P.K.); kkougiou@biol.uoa.gr (K.K.); annakont@upatras.gr (A.K.); 2 Section of Ecology and Systematics, Department of Biology, National and Kapodistrian University of Athens, Panepistimiopolis, 15784 Athens, Greece; ibazos@biol.uoa.gr; 3 Bakkevej 6, DK-5853853 Ørbæk, Denmark; arne.strid@youmail.dk

**Keywords:** taxonomy, phylogeny, functional traits, endemism, chasmophyte, sparsely vegetated land, biodiversity hotspots, mountain flora, GIS analysis, MAES, life-IP 4 natura

## Abstract

Sparsely vegetated habitats of cliffs and screes act as refugia for many regional and local endemic specialized plant taxa most of which have evolved precisely for that type of habitat. The interplay between taxonomic, phylogenetic, and functional plant diversity on rock and scree habitats of extreme environmental conditions, enlightens the relations of plant communities and ecosystems and facilitates management planning for the conservation of biodiversity and ecosystem services. The identification of biodiversity patterns and hotspots (taxonomic, phylogenetic, and functional) contributes to the integration of the ecosystem services (ES) approach for the mapping and assessment of ecosystems and their services (MAES) implementation in Greece and the creation of thematic maps based on the MAES reporting format. The overlap among the protected areas’ network revealed that almost all areas of cliffs and screes of medium, high, and very high taxonomic and phylogenetic plant endemism are included in the Natura 2000 area network. The results of this study provide the baseline information for ES assessments at sparsely vegetated land of cliffs and screes. Our results contribute to the implementation of certain indicators of the national set of MAES indicators in Greece such as (a) floristic diversity and (b) microrefugia of endemic diversity and support of decision-making.

## 1. Introduction

Within the conceptual framework of mapping and assessment of ecosystems and their services (MAES) [1], various studies on ecosystem services (ES) of different ecosystem types, from the local to national level, have been conducted in Greece (e.g., [2,3,4,5,6,7,8,9]). The Life Integrated Project with the acronym “LIFE-IP 4 NATURA”, led by the Hellenic Ministry of Environment and Energy, incorporates MAES implementation Actions at the local and national level [10]. Following the ongoing progress on (a) drafting species’ and habitats’ action plans and area-prioritization efforts inside the Natura 2000 Special Areas of Conservation (SACs) and (b) “LIFE-IP 4 NATURA” actions, the first national, fine-scale, and phylogenetically informed identification of biodiversity hotspots and endemism centers (ECs) has been recently accomplished in Greece [11]. Spatially explicit (i.e., georeferenced) biodiversity information has been obtained and contributed to the development of biodiversity-related indicators for the MAES implementation in Greece [3]. The identified biodiversity hotspots and endemism centers are mainly located on mountainous areas; however, conservation efforts should target areas with overlaps among protected areas and climatic refugia, characterized by high diversity [12,13,14,15,16]. Thus, the preservation of evolutionary heritage, trait diversity, and future ecosystem services for human well-being [17,18,19] could be ensured.

Combined studies of taxonomic and phylogenetic diversity may: (a) reveal the distinct roles of geohistorical and ecological processes in shaping diversity patterns and community structure (b) reveal the mechanisms generating and maintaining biodiversity, such as geographic isolation, environmental filtering, and convergent adaptation [20,21,22,23,24,25] and (c) quantify the evolutionary relatedness of species co-occurring within and across regions [22,26]. Spatial phylogenetics enable the identification of endemism centers and provide valuable insights regarding the ecoevolutionary and conservation value, and the biogeographical origin of a given area [18]. Therefore, identifying biodiversity hotspots and endemism centers and incorporating phylogenetic information is a valuable tool in conservation prioritization and planning [11].

Together with taxonomic and phylogenetic diversity (PD), there is an increasing interest in using functional diversity (FD) to get insights to ecosystem functioning [27], since functional traits account for potential functional redundancy [28] and provide a mechanistic link to observed diversity effects [29]. The combination of the species-based and the functional type-based approaches has the potential to contribute in practical management for the conservation of diversity and ecosystem services [29]. The plant functional trait approach sheds more light to the links of community structure and ecosystem functioning compared to the consideration of species richness alone, since plant functional traits (PFTs) are tightly linked to the microenvironmental conditions [30,31].

Sparsely vegetated land is a MAES level 2 category that includes habitats with sparse vegetation cover due to the prevailing extreme environmental conditions [1]. MAES level 3 category of the sparse vegetation ecosystem type incudes areas of cliffs and screes occurring at medium to high elevations [3,32]. According to EUNIS (European Nature Information System) classification, (a) inland cliffs are unvegetated, sparsely vegetated and bryophyte- or lichen-vegetated, with many endemic plant species and (b) screes are accumulations of boulders, stones, rock fragments, pebbles, gravels, or finer material, of non-aeolian depositional origin, unvegetated, occupied by lichens or mosses, or colonized by sparse but highly specialized plant communities of herbs or shrubs, represented mainly in the high-mountain and mountainous belts [33,34].

Mountainous topography, including cliffs, steep slopes, and screes, favors high endemic species richness [35]; thus, mountains are considered significant “science labs” due to their ecosystems’ high sensitivity to climate change (among others [2,36,37,38]). Habitats with a high-stress level, such as cliffs and screes, are related to the abundance of Mediterranean and Greek endemics and they are dominated by (a) stress tolerator species to harsh environmental conditions, (b) weakly competitive species, and (c) species intolerant to human disturbance [39,40,41].

In the Greek mountains, cliffs and screes are striking geomorphological features that support specialized chasmophytes, scree plant communities, many regional and local endemics, and rare, endangered, and vulnerable taxa [11,19], some of which are relicts of past biogeographic patterns [42]. According to [43], chasmophytic plant communities of Greece are classified in four orders (*Androsacetalia* *vandellii* Br.-Bl. in Meier et Br.-Bl. 1934: on siliceous rocks of high altitudes; *Onosmetalia frutescentis* Quézel 1968: on limestone cliffs of low altitudes; *Potentilletalia speciosae* Quézel 1964: on calcareous rock crevices of high mountain ranges; *Cirsietalia chamaepeuces* Horvat in Horvat, Glavač et Ellenberg ex Bergmeier et al. 2011: on calcareous cliffs of low and mid-altitudes in the Aegean region) within the class *Asplenietea trichomanis* (Br.-Bl. in Meier et Br.-Bl. 1934) Oberd. 1977 (crevices, rocky ledges, and faces of rocky cliffs). Scree plant communities occur on unstable substrata from the meso- to the oro- Mediterranean vegetation belts; these are classified in one order (screes of medium and high altitudes in the Balkans, Crete, and Crimea: *Drypidetalia spinosae* Quézel 1964) within the class *Drypidetea spinosae* Quézel 1964 [42,43,44]. The majority of the characteristic taxa of the rock- and scree-communities are Greek, Aegean, or Cretan endemics; the differentiation of these species-poor plant communities is determined by climate, elevation, geological substrate, aspect, and microhabitat conditions [45,46,47,48,49]. A detailed understanding of the local distribution and the specific habitats of these rare plants provides an opportunity for local conservation efforts that can influence biodiversity conservation at a larger scale [50].

Cliffs and rocky steep slopes promote endemism, through geographical isolation, and are characterized by high ecological specialization and remarkable phytogeographical, genetic, and evolutionary value [51]. In Greece, the high proportion (>65%) of obligate endemic chasmophytes (occurring exclusively on cliffs) indicates a clear correlation of chasmophytic ecology and endemism [50].

Chasmophytes grow in habitats exposed to long periods of hot sunshine, which are characterized by extremely limited soil moisture conditions [40,41] and are more or less inaccessible to herbivores. Due to their habitat, the chasmophytes have many functional characteristics in common: generally long-lived, woody-based perennials with prolonged and conspicuous flowering, high germinability of seeds, and various long-distance seed dispersal mechanisms.

In this context and in the frame of our research project we conducted analyses on cliff- and scree-communities of medium to high altitude mountainous areas of Greece characterized by high endemism rate under the standardized mapping unit of 10 km × 10 km EEA reference grid cell [52] for the national scale MAES implementation reporting scheme for Greece [3,53], with the following objectives: (a) to investigate the biodiversity patterns and locate hotspots of cliffs and screes based on three different diversity facets (taxonomic, phylogenetic, and functional), (b) to identify their ecosystem services based on the different diversity facets, (c) to assess their overlap with the Natura 2000 Network in Greece, (d) to provide baseline information for the MAES implementation on sparsely vegetated land of mountainous cliffs and screes, and (e) to support decision making and policy needs of the National and EU Biodiversity Strategy and the EU Green Deal.

## 2. Results

### 2.1. Plant Taxa of Cliffs and Screes

The plants database occurring (exclusively or non-exclusively) on cliffs and screes of Greece includes 931 native plant species and subspecies assigned to 56 families and 207 genera. Thirty-nine (39) species and subspecies are Pteridophytes belonging to 6 families and 13 genera, 2 are Gymnosperms, and 890 are Angiosperms. The dominance order of the chorological categories decreases as follows: Greek endemic taxa (46.6%), Mediterranean taxa (23.1%), widespread taxa (17.3%), and Balkan taxa (13%). Greek endemics belong to 34 families and 111 genera. Of the total flora registered 54.2% and 67.4% of the endemics prefer exclusively sparsely vegetated areas of cliffs and screes (Figure S1).

Asteraceae is the most taxon-rich family of the total and the endemic flora of cliffs and screes respectively, followed by Caryophyllaceae, Campanulaceae, Lamiaceae, and Rubiaceae (Figure S2a). Asteraceae is also the most genus-rich family of the total and the endemic flora of cliffs and screes respectively, followed by Brassicaceae and Lamiaceae. Campanula is the most taxon-rich genus in cliffs, followed by Hieracium, Silene, Centaurea, and Asperula (Figure S2b).

**Figure 1:**
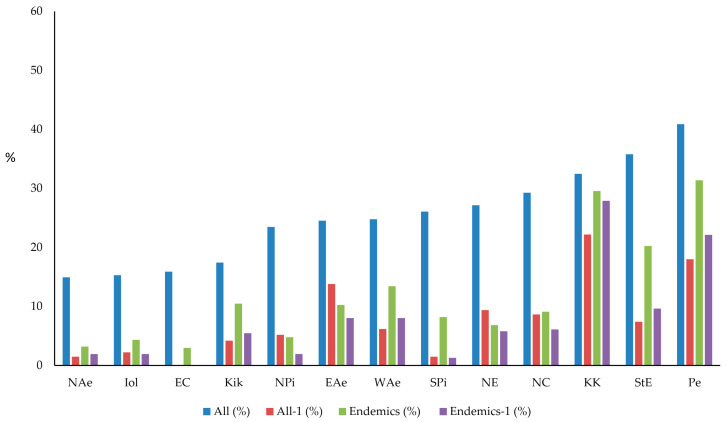
Proportion of the plant taxa (exclusive and non-exclusive) occurring on cliffs and screes of the 13 floristic regions of Greece. Abbreviations: All = proportion of the total flora of cliffs and screes occurring in each floristic region, All-1 = proportion of taxa of the total flora of cliffs and screes occurring in one of the 13 floristic regions, End = proportion of endemic taxa occurring in each floristic region, End-1 = proportion of endemic taxa occurring in one of the 13 floristic regions. Floristic regions: NAe = North Aegean Islands, IoI = Ionian islands, EC = East Central Greece, Kik = Kikladhes (Cyclades), NPi = Northern Pindos, EAe = East Aegean Islands, WAe =West Aegean Islands, SPi = Southern Pindos, NE = North East Greece, NC = North Central Greece, KK = Kriti (Crete)-Karpathos, StE = Sterea Ellas, Pe = Peloponnisos.

Figure 1 shows the distribution of the plant taxa of cliffs and screes of the total and the endemic flora in the 13 floristic regions of Greece. Almost 48% of the total and 71% of the Greek endemic flora are represented by low frequency of occurrence r in one of the 13 floristic regions of Greece. The floristic regions of Pe, StE, and KK are the richest concerning their total and the endemic flora on cliffs and screes, but in reverse order (KK, Pe, and StE) and this is also the case for taxa occurring in only one of the floristic regions.

Regarding the functional traits of the taxa included in our analyses, hemicryptophytes and chamaephytes represent the higher proportions of the life forms (Figure S3), perennials (longevity), caespitose (growth form), and entomogamous (pollination type) taxa dominate, while barochorous, anemochorous, and zoochorous (including epizoochorous, endozoochorous, and myrmecochorous) taxa (dispersal mode) codominate (Figure S3).

### 2.2. Biodiversity Patterns

#### 2.2.1. Taxonomic and Phylogenetic Diversity

**Figure 2:**
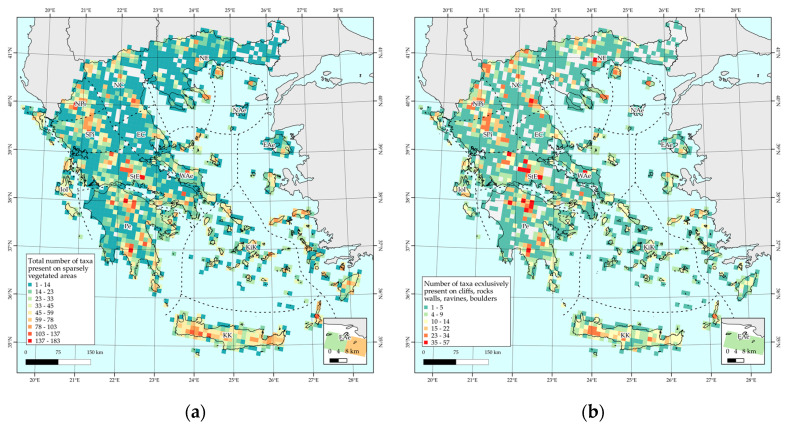
Total number of plant taxa, per 10 km × 10 km EEA reference grid cell: (**a**) present in sparsely vegetated areas and (**b**) exclusively present in sparsely vegetated areas.

Species richness (SR) is generally higher in southern and insular Greece (Pe, StE, KK, and Kik), and near or at several mainland mountain massifs, being highest on Mts. Chelmos and Killini in the Peloponnese (Pe), followed by Mts. Parnassos (StE) and the Vikos gorge (NPi). Several other mountains, such as Mts. Iti, Giona, Timfristos (StE), Olympos (NC), Dirfi (WAe), Taygetos and Parnonas (Pe), and the Pindos mountain range (NPi) display high SR values. This is also true for the vast majority of the large Aegean (KK, Kik, EAe, and NAe floristic regions) and Ionian Islands (IoI), with Crete and Karpathos (KK), Kythira (Pe), Naxos, Andros and Tinos (Kik), Ikaria and Samos (EAe), Skyros (WAe), Thasos, and Samothraki (NAe) standing out, having higher SR values than low- to mid-elevation mainland areas. Figure 2 depicts the thematic representation of the total number of taxa present in cliffs and screes per 10 km × 10 km EEA reference grid.

**Figure 3:**
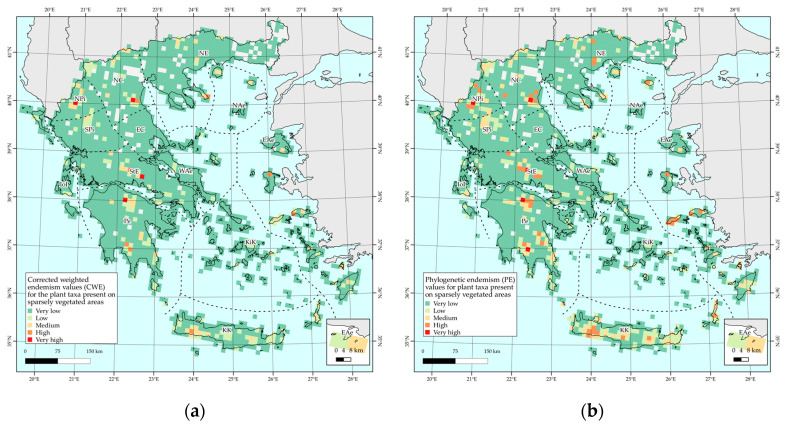
Corrected weighted endemism values (CWE) (left-**a**) and phylogenetic endemism (PE) values (right-**b**) of endemic plant taxa present in sparsely vegetated areas, per 10 km × 10 km EEA reference grid cell.

Areas with very high corrected weighted endemism (CWE) values are mainly found at or near mountain massifs, such as Vikos gorge (NPi), Mt. Olympos (NC), Mt. Chelmos (Pe), Mt. Parnassos (StE), and other mainland mountain ranges, with some cells from, e.g., Crete and Karpathos (KK), Naxos (KiK), Ikaria and Samos (EAe), Samothraki, and Thasos (NAe) exhibiting medium to high CWE values (Figure 3a). Regarding the phylogenetic endemism (PE), it follows approximately the same general trend as the CWE metric: Vikos gorge (NPi), Mt. Olympos (NC), Mt. Giona (StE), Mt. Chelmos, and Mt. Taygetos (Pe) display the highest PE values (Figure 3b).

**Figure 4:**
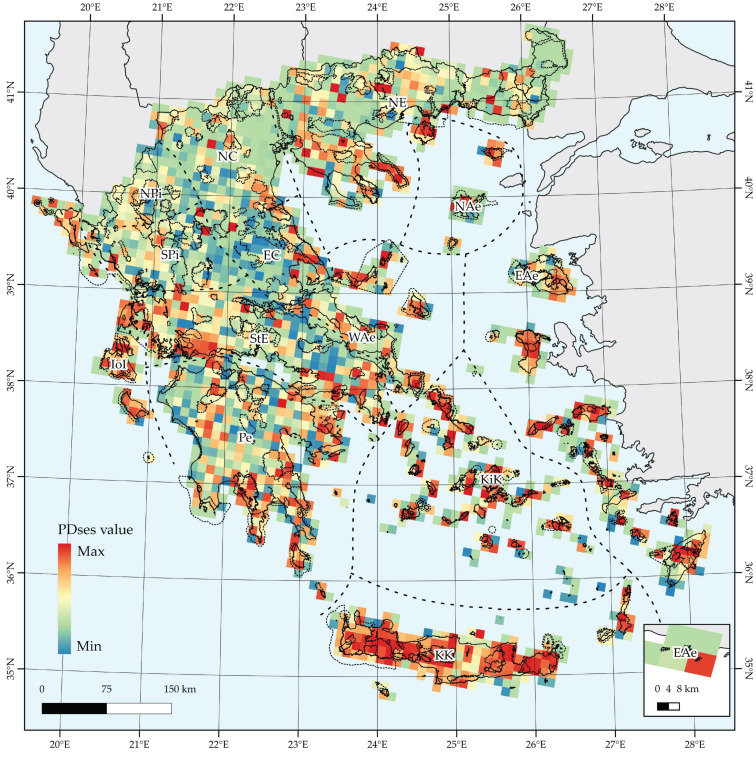
Thematic representation of standardized effect size scores of phylogenetic diversity (PDses) values.

Several areas in Crete and Karpathos (KK), in KiK (Andros, Kea, Syros, Sifnos, Naxos, Paros, Ios, Santorini, and Astypalea), in EAe (Ikaria, Samos, Kos, Nisyros, and Rodos) and NAe (Limnos, Thasos, and Samothraki), and some areas mainly in Southern Peloponnesian (Pe) mountains Taygetos and Parnon, display statistically significant phylogenetic overdispersion (PD_SES_ ≥ 1.96), while most of the Greek territory displays insignificant PD_SES_ values (Figure 4). Statistically significant phylogenetic clustering (PD_SES_ ≤ −1.96) is mainly recorded in some areas in Evvia (WAe), and in some mountainous areas of mainland Greece as Mt. Killini and the central Peloponnesian mountains (Pe), Mts. Parnassos and Giona (StE), and some cells in the northern Pindos mountain range (NPi). Most high-altitude areas in insular and mainland Greece had significantly high CWE values according to the randomization tests, while some Aegean islands displayed significantly low CWE values based on the same tests (Figure S4).

#### 2.2.2. Functional Diversity

The diversity assessment of the selected functional characteristics revealed the following:

- Life form: 22.43% of the 2033 cells are highlighted as the most diverse with five different life forms (Figure S5a). Almost one-fifth of these cells (17.54%) are in the floristic region of Kriti-Karpathos (KK), followed by Pe (15.79%) and StE (14.25%).
- Growth form: A small fraction of the total cells (3.64%) are highlighted as the most diverse with 8–10 different growth forms (Figure S5b). One-fourth of these cells (24.32%) are in the floristic region of StE, followed by Pe (16.22%), NPi and NC (13.51% each).
- Longevity: More than one-third of the total cells (37.58%) are highlighted as the most diverse with three different longevity statuses, i.e., annual, biennial, and perennial (Figure S5c). Nearly one fifth of these cells (18.59%) are located in the floristic region of Pe, followed by KK (16.88%) and StE (14.53%).
- Dispersal mode: More than one-tenth of the total cells (11.02%) are highlighted as the most diverse with 6–7 different dispersal modes, i.e., autochory, barochory, anemochory, and zoochory with epizoochory, endozoochory, and myrmecochory (Figure S5d). Nearly one fifth of these cells (18.30%) are located in the floristic region of Pe, followed by StE (14.29%) and KK (14.29%).
- Pollination type: Almost one-third of the total cells (31.14%) are highlighted as the most diverse with 3-4 different pollination types (Figure S5e). Nearly one fifth of these cells (15.17%) are located in the floristic region of Pe, followed by KK (14.54%) and StE (12.16%).
- Petal color: 27 out of 2033 cells (1.32%) are highlighted as the most diverse with 26–39 different petal colors (Figure S5f). Most of these cells (8 out of 27: 29.63%) are in the floristic region of StE, followed by Pe, SPi, and NPi (5 cells at each: 18.52%).

#### 2.2.3. Distribution and Area of Cliffs and Screes in SACs

**Figure 5:**
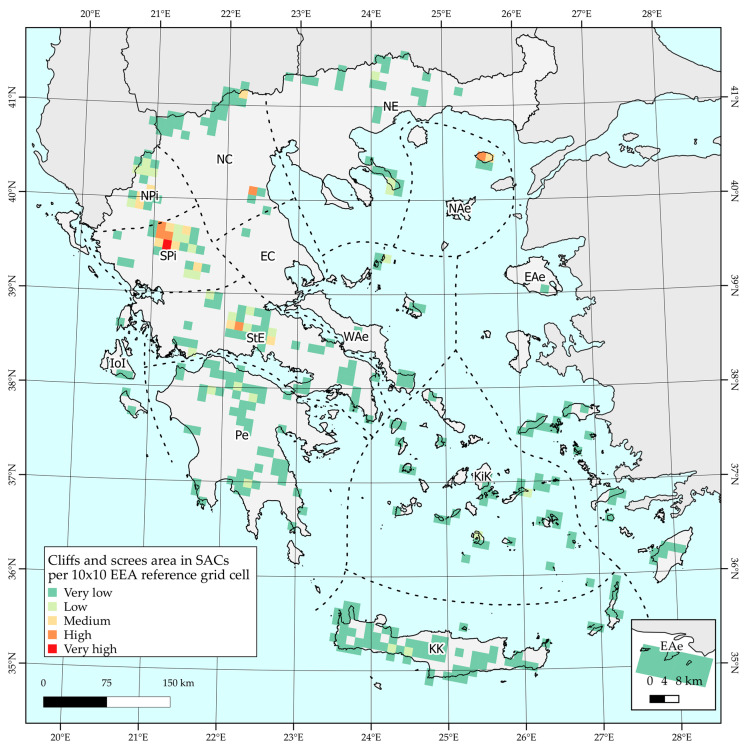
Distribution and area cover in SACs, per 10 km × 10 km EEA reference grid cell.

Cliffs and screes cover ca. 34,904 ha inside the Natura 2000 SACs and are distributed among 127 SACs throughout Greece. The largest areas are located to the Pindos mountain range and especially at Mt Lakmos (GR2130007) and Mt Athamanon (GR2110002); Mts Vardousia (GR2450001), Mt Olympos (GR1250001), and Mt Fengari (GR1110004) follow. Figure 5 provides a thematic representation of the area covered by cliffs and screes in SACs, per 10 km × 10 km EEA reference grid cell.

### 2.3. Ecosystem Services Indicators

For the proposed ecosystem services indicators, the results revealed that eleven (11) ES, at the CICES class level, have been identified as relevant to the selected ES indicators (Table 1): (a) four classes are included in the provisioning (biotic) section, (b) three classes are included in the regulating and maintenance (biotic) section, and (c) five classes are included in the cultural (biotic) section.

### 2.4. Biodiversity Hotspots and Protected Area Network Overlap

**Figure 6:**
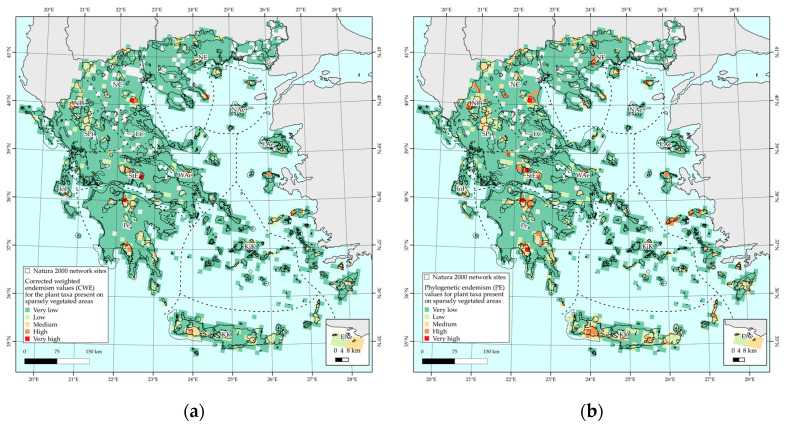
Overlap of the Natura 2000 network sites with corrected weighted endemism values (CWE) (**a**) and phylogenetic endemism (PE) values (**b**) of endemic plant taxa present in sparsely vegetated areas of Greece, per 10 km × 10 km EEA reference grid cell.

The overlap among the protected areas’ network revealed that (a) all cells of medium, high, and very high CWE values are included in the Natura 2000 network (Figure 6a), (b) all cells of very high PE value are included in the Natura 2000 protected area network, while only one cell with high and medium PE value, respectively, is outside the Natura 2000 network, located in the NC floristic region (Figure 6b).

## 3. Discussion

Sparsely vegetated land of cliffs and screes act as reservoirs of relict biodiversity [54], as they have unchanging climates and properties that buffer against climatic fluctuations [41]. Cliffs and screes function as evolutionary dead-ends sites because the future specialization dynamics of any taxon, once it becomes specialized for extreme site conditions, will be favored by very strong selection pressure of growth factors against the biological competition [55]. Cliffs, screes, and rockfalls in collapse dolines are also habitat types in which the impact of abiotic factors on the occurrence and survival of chasmophytes prevail over the biological competition, clearly suggesting that both rock- and scree-dwellers are stress-tolerant against abiotic factors ([56] and references therein).

The combined research of taxonomic, phylogenetic, and functional diversity puts forward challenges to compare their relative roles on ecosystem functioning [57,58,59]. High chasmophytic diversity results in a chasmophytic flora varying along different environmental gradients; temperature can be identified as the factor most strongly correlated with the variation of chasmophytic vegetation [60]. Global warming is severely impacting species distributions and is among the major drivers of species extinctions, since in high-altitude regions an increase in species richness, as a result of upward movement of generalist species, may undergo local extinction of endemic specialists and rare plants such as the endemic obligate chasmophytes ([61,62,63] among others). It has been exemplified in the Cretan highlands [17].

### 3.1. Taxonomic and Phylogenetic Patterns

Total taxon richness follows the same patterns for both floristic-type categories (exclusive- and non-exclusive occurrence) on sparsely vegetated areas of cliffs and screes. This can be explained by the fact that chasmophytic communities are characterized by very low vegetation cover and high endemism rate [50]. This is due to periodic climate change causing the local extinction of some cliff species, but not others, followed by a very slow rate of reinvasion [41]. In general, taxonomic diversity on cliffs and screes is higher in southern and insular Greece, with higher and drier areas being the most taxon rich. Northern phytogeographical regions (NPi and NC) display the highest native richness, while southern phytogeographical regions (StE, Pe, and KK) host most endemic taxa occurring in the sparsely vegetated areas of cliffs and screes. Our results are in line with [11,19], as most of these taxa are concentrated at the Cretan and Peloponnesian mountain massifs. We should note that several central (Naxos, Andros, and Tinos), eastern (Ikaria and Samos), and northern (Thasos and Samothraki) Aegean islands host a high number of obligate cliff endemics and stand out as important SR and CWE hotspots. This is most probably attributed to the extensive cliff system that existed in the Aegean during the quaternary [64] and served as refugia during the quaternary glaciations, thus leading to genetic differentiation and taxonomic isolation. This is in line with our results regarding PD_SES_ and PE, since several Aegean islands exhibit statistically significant phylogenetic overdispersion and display high PE values, suggesting that many distinct and geographically constrained lineages occur there. The same is true for the Southern Peloponnesian mountains (Taygetos and Parnonas), which constitute significant endemism centers [11]. In addition, the Northern Peloponnesian mountains and the mountains of Sterea Ellas and Northern Pindos that show high SR and CWE values are characterized by statistically significant phylogenetic clustering, probably because they have acted as quaternary refugia for cold-adapted species ([11] and references therein). It seems that the incorporation of phylogenetic information in biodiversity analyses can thus reveal—even though indirectly as in our case—the influence of geohistorical and ecological processes on shaping biodiversity patterns [21,22,23,24,25].

### 3.2. Functional Diversity and Ecosystem Services

The functional approach is a powerful tool for identifying the role of biological diversity in the provision of ecosystem services and for predicting their future in a changing world [65]. The use of traits allows for better quantification of ecosystem services and the identification of particular trait–process–service relationships is necessary to establish the biological bases for the current and predicted provision of the ecosystem services under the variable conditions, linked with current global changes [65]. Identifying particular trait–process–service relationships and/or of trade-offs among ecosystem services, is an essential step in establishing mechanistic models for predicting them at the scales of ecosystem and landscape (among others [66,67]).

Most of the plant taxa on cliffs and screes are perennials, hemicryptophytes, and chamaephytes. The characteristics of these life forms enable plants to withstand the coldest winter months under the protection of snow cover [68]. Under the exposed conditions (high wind and frost) of Mediterranean cliffs and screes at higher altitudes, a woody habit probably possesses mechanical advantages and leads to an increase in the bushiness of the plants making cushion chamaephytes abundant [45]. Following life forms adaptations, a large portion of the plant taxa on cliffs and screes’ growth form is caespitose (51%) characterized by short stems and branches that grow in dense tufts or clumps. In total, the most diverse cliffs and screes regarding life forms cover <10% of the mapped cells and regarding growth forms about 27% of the mapped cells. These results confirm that in general, cliffs and screes are mainly characterized by life forms and growth forms that enable plants to survive under the extreme environmental conditions prevailing on these habitats. Growth form composition and diversity including life forms and longevity (i.e., annuals versus perennial, phanerophytes vs. therophytes, and woody versus grasses), and also seed dispersal are considered as ecosystem processes. These ecosystem services affect several others (i.e., soil fertility and nutrient cycling, climate regulation, persistence and resistance of habitat and processes, and biological control) [69,70].

Floral characteristics as petal color together with plant pollination type are related to pollination. White or light-colored plants blend into the background and so they become cryptic to herbivores [71]. The most diverse sparsely vegetated areas of cliffs and screes in Greece, regarding petal color of taxa, cover just 3.7%, showing the specialization of the flowers to certain pollinators and also their strategy against herbivores.

At elevations below 900 m, the peak of the flowering season for communities of chasmophytes is several weeks later than that of adjacent hillside associations and may be due to the conditions of the habitat, but also due to the comparatively late-flowering habit of the families that are most frequently represented in cliff communities [45]. Many of the plant taxa of cliffs and screes, belonging to families such as Asteraceae, Apiaceae, Brassicaceae, Lamiaceae, Hypericaceae, and Valerianaceae are aromatic and/or medicinal (among others, [72,73,74]). Their potential to provide a variety of ES should be assessed, since the importance of aromatic and medicinal plants in various aspects of ecosystem services is highlighted by [72] (exemplified by the Lamiaceae Greek endemics), proposing numerous ES indicators.

The knowledge of when and to what extent rare species can affect ecosystem services is important for identifying situations in which multiple conservation objectives (protecting biodiversity and providing other ecosystem services) are more or less aligned [75]. An option value condition usually refers to preserving a value that has yet to be quantified or even identified. In the context of biodiversity, the importance of species to ecosystem function, and ultimately to goods and services, turns to be apparent through their loss and the impact of this loss on ecosystem services [76]. In Greece, the vast majority of the Greek endemics are considered as threatened and the Cretan (KK) and Peloponnesian (Pe) mountain massifs constitute threatened Greek endemic diversity hotspots [19]. Most of the areas identified herein as biodiversity hotspots coincide with the areas recognized as threatened diversity hotspots in Greece. The areas identified as biodiversity hotspots for cliffs and screes display overlap with the threatened biodiversity hotspots found in Greece since most plant taxa occurring on cliffs and screes are Greek endemics. These areas could be also considered as threatened hotspots.

### 3.3. Ecosystem Services and MAES Implementation

Plant taxa on cliffs and screes are an integral component of sparsely vegetated ecosystems, and the value they provide in terms of services should be included in ecosystem assessments [76]. The subsequent development of proxy indicators, such as plant diversity [9], adds value to the adopted scheme for use and further development of the National Set of MAES Indicators in Greece [3]. Based on the requirements for the MAES implementation in Greece [3,77] and the indicator framework on ecosystem condition assessment [78], the results of the study provide the baseline information for ES assessments at sparsely vegetated land of cliffs and screes and guide thematically and spatially further efforts and assessments. More precisely, the diversity (descriptive and spatial) outcomes contribute to (a) the floristic diversity (code: IB2) and (b) the microrefugia of floristic endemic diversity (code: IB3) indicators of the National Set for the MAES implementation in Greece [3].

Moreover, the analyses and thematic outcomes at the 10 × 10 EEA reference grid comply with the national efforts to provide ES data under a standardized mapping unit for the national scale, facilitating the creation of a national database for the MAES implementation in the country.

## 4. Materials and Methods

The methodological approach employed in this study consists of six main steps: (i) delineation of plant taxa present at sparsely vegetated areas of cliffs and screes, (ii) identification of biodiversity patterns and hotspots (taxonomic, phylogenetic, and functional), (iii) integration of the ecosystem services approach for the MAES implementation in Greece, (iv) creation of thematic maps based on the MAES reporting format [3], (v) interpretation of the overlap with the Natura 2000 terrestrial network, and (vi) provision of management information to support decision making.

### 4.1. Plant Taxa of Cliffs and Screes

Based on the most extensive and detailed database (Flora Hellenica Database) [79,80] and Strid (ongoing) of plants occurring in Greece (1.2 m occurrences), we extracted information on the plant taxa, present at the sparsely vegetated land of cliffs and screes focusing on mountainous areas. These taxa were classified, as either obligate chasmophytes occurring exclusively on cliffs and/or screes or as facultative chasmophytes occurring non-exclusively in these habitats [50,81]. We used a dataset that includes information on chorology and life forms of the taxa, and their geographical distribution in the 13 floristic regions of Greece [40], according to [82,83,84]. The 13 floristic regions are as follows: North East Greece (NE), North Central Greece (NC), East Central Greece (EC), Northern Pindos (NPi), Southern Pindos (SPi), Sterea Ellas (StE), Peloponnisos (Pe), Ionian Islands (IoI), North Aegean Islands (NAe), West Aegean Islands (WAe), East Aegean Islands (EAe), Kiklades (Cyclades) (Kik), and Kriti (Crete)-Karpathos (KK).

All plant names (Table S1) were cross-checked for synonyms, following the nomenclature proposed by [1,2]. More information concerning obligate and facultative chasmophytes of Greece is included in [50,81].

### 4.2. Biodiversity Patterns

Biodiversity patterns are identified based on the 10 km × 10 km EEA reference grid using QGIS 3.14 [85] and following [11] for all spatial analyses. We estimated the species richness (SR) and its geographically-weighted variant (CWE; [86,87,88]) for each grid cell following [11], using functions from [89,90]. As a next step, we used a null model with 999 permutations to assess the statistical significance of the CWE scores for each grid cell, using functions from [87,88]. For the estimation of phylogenetic endemism (PE; the geographically weighted variant of phylogenetic diversity, which corresponds to the total branch length from the dated phylogenetic tree of the lineages present at a grid cell divided by the range sizes of the respective lineages [91]) and the standardized effect size scores of phylogenetic diversity (PD_SES_; [92,93]), we used the time-calibrated tree from [11], keeping only the plant taxa comprising our dataset. We subsequently estimated PE and PD_SES_ (see the Supplementary Material for more details on this metric) for each grid cell using functions from the ‘phyloregion’ 1.0.4 [94,95,96] and the ‘PhyloMeasures’ 2.1 [97] R packages, respectively. Afterwards, following [11,98], we located the biodiversity hotspots for all taxonomic and phylogenetic biodiversity metrics that correspond to the highest 1% values (L1) for each of these metrics. We identified these biodiversity hotspots using functions from the ‘phyloregion’ 1.0.4 [94,95,96] R package. Biodiversity hotspots are herein and hereafter defined as regional biodiversity hotspots (i.e., hotspots within global biodiversity hotspots [99]).

Regarding functional traits identification and delineation, we used data available for a subset of the taxa included in our analyses (*n* = 267) (Table S2). Functional traits used in this study concerning life form, longevity, growth form, petal color, pollination type, and dispersal mode were derived from botanical descriptions [79,80,100,101,102,103] and the Baseflore database (http://perso.wanadoo.fr/philippe.julve/catminat.htm, accessed on 10 December 2020), focusing on taxa occurring in mid- to high-altitudes.

### 4.3. Ecosystem Services

Based on the MAES (mapping and assessment of the ecosystem and their services) approach [1,78] and the provisions for the development of MAES indicators for Greece [3], we used taxonomic, phylogenetic, and functional diversity to propose MAES related indicators for the cliffs and screes included in sparsely vegetated land ecosystem type [3,32]. The delineation of ecosystem services follows the Common International Classification of Ecosystem Services (CICES) (https://cices.eu/, accessed on 10 February 2021). More precisely, the different facets of biodiversity are assigned to one or more ES categories following [65,69,104,105,106]. Among plant traits having a major contribution to the provision of numerous ES are life form, longevity, growth form, petal color, pollination type, and dispersal mode [21,65,106]. Some species present more than one type of the same trait.

### 4.4. Priority Hotspots and Protected Area Overlap

We followed the rationale proposed by [11] regarding the identification of priority hotspots and as such, we used the CWE and PE metrics for this purpose, which are more robust when locating biodiversity hotspots [84,85,86]. Thereafter, we overlapped L1 hotspot results with the protected areas (PAs) network retrieved from the World Database on Protected Areas (WPDA) (i.e., the Natura 2000 network sites), using GIS-related functions from the “wdpar” 1.0.0 [107] and the “sf” 0.8.0 [108] R packages, to assess the effectiveness of the protected area network in Greece.

### 4.5. Thematic Mapping

The results are presented in thematic maps created in QGIS [83], using the 10 km × 10 km EEA reference grid [52] as the mapping unit, supporting the national efforts for a standardized reporting of all MAES related information and data at the national scale.

## Figures and Tables

**Table 1:** Correspondence of the different facets of biodiversity identified for the plants of cliffs and screes, based on the CICES system.

Section	Class	Code	Relevant ES Indicators
Provisioning (biotic)	Fibers and other materials from wild plants for direct use or processing (excluding genetic materials)	1.1.5.2	G, L
	Seeds, spores and other plant materials collected for maintaining or establishing a population	1.2.1.1	G, L
	Higher and lower plants (whole organisms) used to breed new strains or varieties	1.2.1.2	T, P
	Individual genes extracted from higher and lower plants for the design and construction of new biological entities	1.2.1.3	T, P
Regulating and maintenance (biotic)	Pollination (or ‘gamete’ dispersal in a marine context)	2.2.2.1	D, C, PL
	Maintaining nursery populations and habitats (including gene pool protection)	2.2.2.3	T, P, G, D
	Control of erosion rates	2.2.1.1	G, L, LV
Cultural (biotic)	Characteristics of living systems that enable activities promoting health, recuperation, or enjoyment through passive or observational interactions	3.1.1.2	T, P, G, L, C, PL
	Characteristics of living systems that enable scientific investigation or the creation of traditional ecological knowledge	3.1.2.1	T, P, PL
	Characteristics of living systems that enable education and training	3.1.2.2	T, P, G, L, D, C
	Characteristics of living systems that enable aesthetic experiences	3.1.2.4	G, L, D, C

T: taxonomic diversity; P: phylogenetic diversity; L: life form; G: growth form; LV: longevity; D: dispersal mode; PL: pollination type; C: petal color.

## Supplementary Materials

The following are available online at https://www.mdpi.com/article/10.3390/plants10050992/s1, Figure S1. Numbers of families, genera, species, and subspecies (exclusive and non-exclusive) preferring sparsely vegetated areas of cliffs and screes. Figure S2: (a) The most taxon-rich families of the floristic composition of cliffs and screes. (b) The most taxon-rich genera of the floristic composition of cliffs and screes. All = total flora of cliffs and screes ((exclusive and non-exclusive). Figure S3: Proportions of different categories of plant traits: life form, longevity, growth form, pollination type, petal color, and dispersal mode. Figure S4: Randomization results (999 runs) for the corrected weighted endemism (CWE) metric. Red coloring indicates areas with statistically significantly higher than expected corrected weighted endemic richness. Blue coloring indicates areas where the CWE values are not statistically significantly higher or lower than expected. Figure S5: Functional diversity patterns for: life form (a), growth form (b), longevity (c), dispersal mode (d), pollination type (e), and petal color (f), per 10 km × 10 km EEA reference grid cell. Table S1: Plant taxa occurring on cliffs and screes and their geographical distribution on the 13 floristic regions of Greece. Table S2: Plant taxa occurring on cliffs and screes in mid- to high-altitudes and their functional traits concerning life form, longevity, growth form, petal color, pollination type, and dispersal mode.
