# Supplementary material for: Linking Taxonomic, Phylogenetic and Functional Plant Diversity with Ecosystem Services of Cliffs and Screes in Greece
Source: Plants (Basel). 2021 May 17;10(5):992. doi: 10.3390/plants10050992 (PMC8156371; doi:10.3390/plants10050992)
Supplement: Supplementary file 1 [file plants-10-00992-s001.zip › Supplementary file Table S1.pdf]

Linking Taxonomic, Phylogenetic and Functional plant diversity with Ecosystem Services of cliffs and screes in Greece

Maria Panitsa*, Ioannis P. Kokkoris, Konstantinos Kougioumoutzis, Anna Kontopanou, Ioannis Bazos, Arne Strid and Panayotis Dimopoulos*

Supplementary file

Table S1. Plant taxa occurring on cliffs and screes and their geographical distribution on the 13 floristic regions of Greece according Dimopoulos et al (2013, 2016) and http://portal.cybertaxonomy.org/flora-greece/ (Dimopoulos et al 2020).

Taxon	IoI	NPi	SPi	Pe	StE	EC	NC	NE	NAe	WAe	Kik	KK	EAe	Chorology	Life form
Achillea abrotanoides (Vis.) Vis.		x												Bk	C
Achillea aegyptiaca L.											x			*	H
Achillea ageratifolia (Sm.) Benth. & Hook. f. subsp. *ageratifolia*					x		x	x						Bk	H
Achillea ageratifolia subsp. *aizoon* (Griseb.) Heimerl							x							Bk	H
Achillea ambrosiaca (Boiss. & Heldr.) Boiss.							x							*	C
Achillea cretica L.											x	x	x	EM	C
Achillea fraasii Sch. Bip.		x	x	x	x	x	x							BA	H
Achillea holosericea Sm.		x	x	x	x	x	x	x		x				Bk	H
Achillea occulta Constantin. & Kalpoutz.				x										*	H
Achillea pindicola Hausskn.			x		x									Bk	H
Achillea pindicola Hausskn. subsp. *pindicola*			x											*	H
Achillea pindicola subsp. *integrifolia* (Halácsy) Franzén			x		x									*	H
Achillea taygetea Boiss. & Heldr.				x										*	H
Achillea umbellata Sm.			x	x						x				*	C
Acinos alpinus (L.) Moench	x	x	x	x	x	x	x	x	x	x	x	x	x	Eu	H
Adiantum capillus-veneris L.	x	x	x	x	x	x	x	x	x	x	x	x	x	ST	G
Aegonychon goulandriorum (Rech. f.) Holub		x	x											*	H
Aetheorhiza bulbosa (L.) Cass.	x	x	x	x	x	x	x	x	x	x	x	x	x	Me	G
Aethionema retsina Phitos & Snogerup										x				*	C
Aethionema saxatile (L.) R. Br.	x	x	x	x	x	x	x	x	x	x	x	x	x	ME	C
Alcea heldreichii (Boiss.) Boiss.	x	x	x		x		x	x	x	x			x	BA	H
Alchemilla amphiargyrea Buser			x		x									Bk	H

Alchemilla aroanica (Buser) Rothm.				x										*	H
Alchemilla velebitica (Janch.) Degen		x	x											Bk	H
Allium aegilicum Tzanoud.				x										*	G
Allium bourgeaui Rech. f.				x	x						x	x	x	EM	G
Allium bourgeaui Rech. f. subsp. *bourgeaui*												x	x	EM	G
Allium bourgeaui subsp. *creticum* Bothmer												x		*	G
Allium bourgeaui subsp. *cycladicum* Bothmer				x	x						x		x	EM	G
Allium brulloi Salmeri											x			*	G
Allium calamarophilon Phitos & Tzanoud.										x				*	G
Allium callimischon Link	x	x	x	x	x							x		EM	G
Allium callimischon subsp. *haemostictum* Stearn												x		EM	G
Allium dilatatum Zahar.												x		*	G
Allium frigidum Boiss. & Heldr.			x	x										*	G
Allium heldreichii Boiss.			x		x	x	x							*	G
Allium platakisii Tzanoud. & Kypriot.												x		*	G
Allium sandrasicum Kollmann & al.													x	EM	G
Allium subhirsutum L.	x		x	x	x	x	x	x	x	x	x	x	x	Me	G
Allosorus acrosticus (Balb.) Christenh.	x	x	x	x	x	x	x	x	x	x	x	x	x	Me	G
Allosorus guanchicus (Bolle) Christenh.				x									x	Me	G
Allosorus persicus (Bory) Christenh.		x	x	x	x		x	x	x	x		x	x	Me	G
Allosorus pteridioides (Reichard) Christenh.	x			x	x			x			x	x	x	MS	G
Alopecurus gerardii Vill.		x	x	x	x		x	x		x				Me	G
Alyssoides cretica (L.) Medik.											x	x		*	C
Alyssum montanum L.	x	x	x	x	x	x	x	x	x	x				EA	C
Alyssum samium T.R. Dudley & Christod.													x	*	H
Amelanchier ovalis Medik.	x	x	x	x	x	x	x	x	x	x		x		EA	P
Amelanchier ovalis Medik. subsp. *ovalis*	x	x			x	x	x	x		x				ME	P
Amelanchier ovalis subsp. *cretica* (Willd.) Maire & Petitm.		x	x	x	x		x			x		x		Me	P
Amelanchier parviflora Boiss.				x	x					x			x	EM	P
Amelanchier parviflora subsp. *chelmea* (Halácsy) Ziel.				x	x					x				*	P
Amelanchier parviflora subsp. *dentata* (Boiss.) K.I. Chr.													x	EM	P
Anogramma leptophylla (L.) Link	x	x	x	x	x	x	x	x	x	x	x	x	x	Co	T
Anthemis ammanthus Greuter											x	x		*	T
Anthemis ammanthus Greuter subsp. *ammanthus*											x	x		*	T

Anthemis ammanthus subsp. *paleacea* Greuter												x		*	T
Anthemis cretica L.	x	x	x	x	x	x	x	x	x	x		x	x	ME	H
Anthemis cretica L. subsp. *cretica*			x	?	x	x	x	?	?	x				ME	H
Anthemis cretica subsp. *leucanthemoides* (Boiss.) Grierson												x	x	EM	H
Anthemis samariensis Turland											x			*	H
Anthemis spruneri Boiss. & Heldr.				?	x	?								*	H
Anthyllis aurea Host		x					x	x						Bk	C
Anthyllis splendens Willd.											x	x		*	H
Aquilegia ottonis Boiss.				x	x		x							Bk	H
Aquilegia ottonis Boiss. subsp. *ottonis*				x	x									*	H
Aquilegia ottonis subsp. *amaliae* (Boiss.) Strid							x							Bk	H
Arabis alpina L.	x	x	x	x	x	x	x	x		x		x	x	ES	H
Arabis bryoides Boiss.				x	x		x	x	x					Bk	H
Arenaria conferta Boiss.		x	x	x	x		x			x				Bk	T
Arenaria cretica Spreng.		?		x	x		x	x				x		Bk	C
Arenaria deflexa Decne.									x		x		x	EM	H
Arenaria deflexa Decne. subsp. *deflexa*											x		x	EM	H
Arenaria deflexa subsp. *microsepala* McNeill													x	EM	H
Arenaria filicaulis Fenzl		x		x	x			x	x	x		x		BA	H C
Arenaria filicaulis Fenzl subsp. *filicaulis*				x	x			x	x					BA	C H
Arenaria filicaulis subsp. *graeca* (Boiss.) McNeill		x		x	x			x		x		x		BA	C
Arenaria filicaulis subsp. *teddii* (Turrill) Strid								x						*	C H
*Arenaria fragillim*a Rech. f.												x		*	C
Arenaria gionae Gustavsson					x									*	H
Arenaria graveolens Schreb.					?					x		?	x	EM	T
Arenaria luschanii McNeill													x	EM	T
Arenaria muralis (Link) Spreng.				x	x				?	x	x	x	x	EM	T
Arenaria rhodia Boiss.													x	EM	T
Arenaria runemarkii Phitos													x	*	T
Armeria icarica J.R. Edm.													x	*	H
Asperula arcadiensis Sims				x										*	C
Asperula aristata L. f.	x	x	x	x	x	x	x	x	x	x				ME	H
Asperula aristata subsp. *condensata* (Boiss.) Ehrend. & Krendl	x	x	x	x	x			x						Bk	H
Asperula baenitzii Boiss.					x									*	H

Asperula boissieri Boiss.				x	x									*	C
Asperula boryana (Walp.) Ehrend.				x										*	C
Asperula chlorantha Boiss. & Heldr.	x	x	x	x	x									Bk	C
Asperula crassula Greuter & Zaffran												x		*	C
Asperula doerfleri Wettst.		x					x							Bk	C
Asperula elonea Iatroú & Georgiadis				x										*	C
Asperula lutea Sm.			x	x	x					x				*	H
Asperula lutea Sm. subsp. *lutea*			x	x	x									*	C
Asperula lutea subsp. *euboea* Ehrend.										x				*	C
Asperula lutea subsp. *griseola* Greuter				x										*	C
Asperula lutea subsp. *mungieri* (Boiss. & Heldr.) Maire & Petitm.				x										*	C
Asperula muscosa Boiss. & Heldr.							x							*	H
Asperula naufraga Ehrend. & Gutermann	x													*	C
Asperula nitida subsp. *mytilinica* Ehrend.													x	*	C
Asperula oetaea (Boiss.) Halácsy			x	x	x									*	C
Asperula ophiolitica Ehrend.										x				*	C
Asperula pubescens (Willd.) Ehrend. & Schönb.-Tem.												x		*	C
Asperula pulvinaris (Boiss.) Boiss.					x									*	C
Asperula purpurea (L.) Ehrend.		x			x		x	x	x	x				ME	C
Asperula purpurea (L.) Ehrend. subsp. *purpurea*							x	x	x					ME	C
Asperula purpurea subsp. *apiculata* (Sm.) Ehrend.		x	x		x		x	x	x					BA	C
Asperula samia Christod. & T. Georgiadis													x	*	C
Asperula saxicola Ehrend.				x										*	C
Asperula suberosa Sm.								x						Bk	C
Asperula suffruticosa Boiss. & Heldr.										x				*	C
Asperula taygetea Boiss. & Heldr.				x								x		*	C
Asperula tournefortii Spreng.											x	x	x	EM	C
Asplenium adiantum-nigrum L.	x	x	x	x	x	x	x	x	x	x	x	x	x	Pt	H
Asplenium aegaeum Lovis & al.					?							x		EM	H
Asplenium bourgaei Milde												x	x	EM	H
Asplenium ceterach L.	x	x	x	x	x	x	x	x	x	x	x	x	x	EA	H
Asplenium creticum Lovis & al.												x		*	H
Asplenium cuneifolium Viv.		x	x		x		x							Eu	H
Asplenium fissum Willd.		x	x	x	x		x	x						Eu	H

Asplenium lepidum C. Presl subsp. *lepidum*			x		x		x					x		Eu	H
Asplenium lepidum subsp. *haussknechtii* (Godet & Reut.) Brownsey												x		MS	H
Asplenium obovatum Viv.				x			x	x	x	x	x	x	x	Me	H
Asplenium petrarchae (Guérin) DC.				x										Me	H
Asplenium ruta-muraria L.	x	x	x	x	x	x	x	x	x	x	x	x	x	Ct	H
Asplenium scolopendrium L.	x	x	x	x	x	x	x	x	x	x	x	x		Pt	H
Asplenium scolopendrium subsp. *antri-jovis* (Kümmerle) Brownsey & Jermy											x	x		EM	H
Asplenium septentrionale (L.) Hoffm.		x	x	x	x	x	x	x	x					Bo	H
Asplenium trichomanes L. subsp. *trichomanes*	x	x	x	x	x	x	x	x	x	x				Co	H
Asplenium trichomanes subsp. *inexpectans* Lovis		x	x	x	x		x		x			x	x	Eu	H
Asplenium trichomanes subsp. *pachyrachis* (H. Christ) Lovis & Reichst.							x					x		Eu	H
Asplenium trichomanes subsp. *quadrivalens* D.E. Mey.	x	x	x	x	x	x	x	x	x	x	x	x	x	EA	H
Asplenium viride Huds.		x	x	x	x		x	x		x		x		Bo	H
Astragalus creticus Lam.												x		*	C
Astragalus mayeri Micevski					x	x	x							Bk	C
Asyneuma giganteum (Boiss.) Bornm.												x	x	*	H
Asyneuma limonifolium (L.) Janch.	x	x	x	x	x	x	x	x	x	x			x	Me	H
Athamanta densa Boiss. & Orph.					x		x							Bk	H
Aubrieta deltoidea (L.) DC.	x	x	x	x	x	x	x	x	x	x	x	x	x	Me	C
Aubrieta erubescens Griseb.								x						*	H
Aubrieta scyria Halácsy										x				*	H
Aubrieta thessala Boissieu							x							*	H
Aurinia moreana Tzanoud. & Iatroú				x										*	H
Aurinia saxatilis (L.) Desv.	x	x	x	x	x	x	x	x	x	x	x	x	x	ME	H
Aurinia saxatilis subsp. *megalocarpa* (Hausskn.) T.R. Dudley	x			x	?						x	x	x	BI	H
Aurinia saxatilis subsp. *orientalis* (Ard.) T.R. Dudley	x	x	x	x	x	x	x	x	x	x			x	BA	H
Ballota glandulosissima Hub.-Mor. & Patzak													x	EM	C
Bellevalia sitiaca Kypriot. & Tzanoud.												x		*	G
Berberis cretica L.			x	x		x	x	x	x	x		x	x	EM	P
Bolanthus laconicus (Boiss.) Barkoudah				x										*	C
Bornmuellera baldaccii (Degen) Heywood		x												Bk	H
Brassica cretica Lam.	x		x	x	x	x		x	x	x	x	x	x	EM	C
Brassica cretica Lam. subsp. *cretica*	x		x	x	x	x		x	x	x	x	x	x	EM	C
Brassica cretica subsp. *aegaea* (Heldr. & Halácsy) Snogerup & al.				x								x		*	C

Brassica cretica subsp. *laconica* M.A. Gust. & Snogerup				x										*	C
Brassica incana Ten.	x													BI	C
Bromus tomentellus Boiss.												x		MS	H
Bubon arachnoideum (Boiss. & Orph.) Hand				x										*	H
Bubon macedonicum L.	x	x	x	x	x					x	x	x		BI	H
Bupleurum kakiskalae Greuter												x		*	C
Bupleurum trichopodum Boiss. & Spruner	x		x	x	x	x	x	x	x	x	x	x	x	Me	T
Calamagrostis varia (Schrad.) Host		x			x		x	x						Eu	H
Calamintha cretica (L.) Lam.												x		*	C
Campanula aizoides Greuter				x								x		*	H
Campanula aizoon Boiss. & Spruner					x									*	H
Campanula anchusiflora Sm.				x										*	H
Campanula andrewsii A. DC.				x										*	H
Campanula andrewsii A. DC. subsp. *andrewsii*				x										*	H
Campanula andrewsii subsp. *hirsutula* Phitos				x										*	H
Campanula asperuloides (Boiss. & Orph.) Engl.				x										*	H
Campanula calaminthifolia Lam.											x			*	H
Campanula carpatha Halácsy												x		*	H
Campanula celsii A. DC.				x	x	x				x	x			*	H
Campanula celsii A. DC. subsp. *celsii*										x				*	H
Campanula celsii subsp. *carystea* Phitos				x	x	x					x			*	H
Campanula celsii subsp. *parnesia* Phitos					x									*	H
Campanula celsii subsp. *spathulifolia* (Turrill) Phitos				x	x									*	H
Campanula columnaris Contandr. & al.					x									*	H
Campanula constantini Beauverd & Topali										x				*	H
Campanula cretica (A. DC.) D. Dietr.												x		*	H
Campanula cymaea Phitos										x				*	H
Campanula cymbalaria Sm.													x	EM	H
Campanula delicatula Boiss.											x	x	x	EM	T
Campanula euboica Phitos					x					x				*	H
Campanula formanekiana Degen & Dörfl.							x							Bk	H
Campanula garganica subsp. *acarnanica* (Damboldt) Damboldt					x									*	H
Campanula garganica subsp. *cephallenica* (Feer) Hayek	x													*	H
Campanula garganica Ten.	x				x									BI	H

Campanula goulimyi Turrill								x				*	H
Campanula hagielia Boiss.											x	EM	H
Campanula hawkinsiana Hausskn. & Heldr.	x		x		x							Bk	H
Campanula heterophylla L.									x			*	H
Campanula hierapetrae Rech. f.										x		*	G
Campanula incurva A. DC.				x	x			x			x	*	H
Campanula jacquinii (Sieber) A. DC.										x		*	H
Campanula laciniata L.									x	x		*	H
Campanula lavrensis (Tocl & Rohlena) Phitos						x	x					*	H
Campanula lyrata Lam.											x	EM	H
Campanula lyrata Lam. subsp. *lyrata*											x	EM	H
Campanula lyrata subsp. *icarica* Phitos											x	*	H
Campanula merxmuelleri Phitos								x			x	*	H
Campanula nisyria Papatsou & Phitos											x	*	H
Campanula oreadum Boiss. & Heldr.					x							*	H
Campanula orphanidea Boiss.						x						Bk	H
Campanula papillosa Halácsy		x										*	H
Campanula pelia Bedd.				x	x							*	H
Campanula pelviformis Lam.										x		*	H
Campanula pinatzii Greuter & Phitos										x		*	T
Campanula rechingeri Phitos								x				*	H
Campanula reiseri Halácsy								x	x			*	H
Campanula rhodensis A. DC.											x	*	T
Campanula rumeliana (Hampe) Vatke					x	x	x	x				BA	H
Campanula rupestris Sm.			x									*	H
Campanula rupicola Boiss. & Spruner			x									*	H
Campanula samothracica (Degen) Greuter & Burdet							x	x				*	H
Campanula saonissia Biel & Kit Tan							x					*	T
Campanula sartorii Boiss. & Heldr.									x			*	H
Campanula saxatilis L.		x								x		*	H
Campanula saxatilis L. subsp. *saxatilis*										x		*	H
Campanula saxatilis subsp. *cytherea* Rech. f. & Phitos		x										*	H
Campanula sciathia Phitos								x				*	H
Campanula scopelia Phitos								x				*	H

Campanula simulans Carlström													x	EM	T
Campanula topaliana Beauverd				x	x									*	T H
Campanula topaliana Beauverd subsp. *topaliana*				x	x									*	T H
Campanula topaliana subsp. *delphica* Phitos					x									*	T H
Campanula tubulosa Lam.												x		*	H
Campanula versicolor Andrews	x	x	x	x	x	x	x	x						BI	H
Campanula wanneri Rochel							?	x						Bk	H
Capparis orientalis Veill.	x		x	x	x	x			x	x	x	x	x	Me	C P
Cardamine calliphaea Kit Tan, Vold & Giannop.				x	x									*	T
Cardamine carnosa Waldst. & Kit.		x	x	x			x							Bk	H
Cardamine glauca DC.		x			x		x	x						BI	T
Carduus tmoleus Boiss.		x	x	x	x	x	x	x	x	x				BA	H
Carlina diae (Rech. f.) Meusel & Kästner												x		*	H
Carthamus rechingeri P.H. Davis												x		*	T
Carum depressum Hartvig & Kit Tan				x										*	H
Carum graecum Boiss. & Heldr.		x	x	x	x	x	x			x				BA	H
*Carum heldreichi*i Boiss.			x	x	x		x			x				ME	H
Celtis planchoniana K.I. Chr.								x						MS	P
Celtis tournefortii Lam.			x	x							x	x	x	Me	P
Centaurea acicularis Sm.													x	*	H
Centaurea argentea L.				x								x		*	H
Centaurea atropurpurea Olivier											x			*	H
Centaurea chrysocephala Phitos & T. Georgiadis		x	x											*	H
Centaurea cuneifolia Sm.		x	x			x	x	x	x					BA	H
Centaurea cuneifolia Sm. subsp. *cuneifolia*							x	x	x					BA	H
Centaurea deustiformis Adamović		x			x		x	x						Bk	H
Centaurea ebenoides S. Moore										x				*	H
Centaurea graeca Griseb.		x	x		x	x	x	x						Bk	H
Centaurea greuteri E. Gamal-Eldin & Wagenitz								x						*	H
Centaurea heldreichii Halácsy					x									*	H
Centaurea huljakii J. Wagner								x						*	H
Centaurea incompleta Halácsy							x							*	H
Centaurea johnseniana Strid & Kit Tan							x	x						*	H
Centaurea kalambakensis Freyn & Sint.			x											*	H

Centaurea laconica Boiss.				x							x			*	H
Centaurea laconica Boiss. subsp. *laconica*				x										*	H
Centaurea lactucifolia Boiss.													x	*	H
Centaurea lancifolia Spreng.												x		*	H
Centaurea leonidia Kalpoutz. & Constantin.				x										*	H
Centaurea litochorea T. Georgiadis & Phitos							x							*	H
Centaurea musakii T. Georgiadis			x											*	H
Centaurea musarum Boiss. & Orph.					x									*	H
Centaurea niederi Heldr.				x	x									*	H
Centaurea pawlowskii Phitos & Damboldt		x												Bk	H
Centaurea peucedanifolia Boiss. & Orph.								x						*	H
Centaurea poculatoris Greuter												x		*	H
Centaurea princeps Boiss. & Heldr.			x		x									*	H
Centaurea rechingeri Phitos										x			x	*	H
Centaurea redempta Heldr.				x								x		*	H
Centaurea redempta Heldr. subsp. *redempta*												x		*	H
Centaurea redempta subsp. *cytherea* (Rech. f.) Routsi & T. Georgiadis				x										*	H
Centaurea rutifolia Sm.								x						Bk	H
Centaurea samothracica Strid & Kit Tan									x					*	H
Centaurea thasia Hayek									x					*	H
Centaurea vermia Rech. f.							x							*	H
Centaurea wettsteinii Degen & Dörfl.							x							*	H
Centaurea xylobasis Rech. f.													x	*	C
Centranthus longiflorus Steven		x	x	x	x		x							MS	H
Centranthus longiflorus subsp. *junceus* (Boiss. & Heldr.) I. Richardson		x	x	x	x		x			x				Bk	H
Centranthus ruber (L.) DC.	x	x	x	x	x	x	x	x	x	x	x	x	x	Me	H
Centranthus ruber subsp. *sibthorpii* (Boiss.) Hayek	x	x	x	x	x	x	x	x		x	x			Bk	H
Cephalaria squamiflora (Sieber) Greuter										x	x	x	x	Me	C
Cerastium candidissimum Correns	x	x	x	x	x	x				x				*	H
Cerastium runemarkii Möschl & Rech. f.										x	x			*	T
Chaenorhinum origanifolium (L.) Kostel.					x									MA	H
Chaenorhinum rubrifolium (DC.) Fourr.				x	x	x	x	x	x			x		Me	T
Cirsium heldreichii Halácsy		x	x	x	x					x			x	*	H
Cirsium morinifolium Boiss. & Heldr.												x		*	H

Cleome iberica DC.													x	EA	T
Clinopodium taygeteum (P.H. Davis) Bräuchler & Heubl				x										*	H
Convolvulus argyrothamnos Greuter												x		*	C
Convolvulus oleifolius Desr.	?		?	x	x	x			x	x	x	x	x	Me	C
Cosentinia vellea (Aiton) Tod.	x			x	x			x		x	x	x	x	MS	G
Cotoneaster creticus J. Fryer & B. Hylmö												x		*	P
Cotoneaster integerrimus Medik.	x	x	x	x	x	x	x	x						EA	P
Cotoneaster nummularius Fisch. & C.A. Mey.													x	MS	P
Cotoneaster tomentosus (Aiton) Lindl.		x	x	x	x	x	x	x	x	x				ME	P
Crepis auriculifolia Spreng.												x		*	H
Crepis heldreichiana (Kuntze) Greuter				x										*	H
Crepis neglecta subsp. *graeca* (Vierh.) Rech. f.			x	x	x	x	x		x	x	x			*	T
Cyclamen graecum Link	x	x	x	x	x	x	x	x		x	x	x	x	EM	G
Cymbalaria acutiloba subsp. *paradoxa* (Greuter) Carcinero & al.													x	*	H C
Cymbalaria longipes (Boiss. & Heldr.) A. Cheval.				x	x					x	x	x	x	EM	H C
Cymbalaria microcalyx (Boiss.) Wettst.	x		x	x	x						x	x	x	Bk	H C
Cymbalaria microcalyx (Boiss.) Wettst. subsp. *microcalyx*				x										*	H C
Cymbalaria microcalyx subsp. *heterosepal*a (Cufod.) Speta				x								x		*	H C
Cymbalaria minor (Cufod.) Speta subsp. *minor*	x		x	x	x									BA	H C
Cymbalaria minor subsp. *dodekanesi* (Greuter) Carcinero & al.											x	x	x	*	H C
Cystopteris alpina (Lam.) Desv.			x		x		x		x					EA	G
Cystopteris fragilis (L.) Bernh.	x	x	x	x	x	x	x	x	x	x	x	x	x	Co	G
Danthoniastrum compactum (Boiss. & Heldr.) Holub		x	x	x	x			x						Bk	H
Daphne jasminea Sm.				x	x					x		x		Me	C
Daphne oleoides Schreb.	x	x	x	x	x	x	x	x		x		x		Me	C
Dianthus cinnamomeus Sm.											x	x		*	H
Dianthus cinnamomeus Sm. subsp. *cinnamomeus*											x	x		*	H
Dianthus cinnamomeus subsp. *naxensis* Runemark											x			*	H
Dianthus desideratus Strid										x				*	C
*Dianthus elegan*s d'Urv.													x	EM	H
Dianthus fruticosus L.	x			x							x	x	x	*	C
Dianthus fruticosus L. subsp. *fruticosus*											x			*	C
Dianthus fruticosus subsp. *amorginus* Runemark											x	x		*	C
*Dianthus fruticosu*s subsp. *carpathus* Runemark												x		*	C

Dianthus fruticosus subsp. *creticus* (Tausch) Runemark												x		*	C
Dianthus fruticosus subsp. *karavius* Runemark													x	*	C
Dianthus fruticosus subsp. *occidentalis* Runemark	x			x								x		*	C
Dianthus fruticosus subsp. *rhodius* (Rech. f.) Runemark													x	*	C
Dianthus fruticosus subsp. *sitiacus* Runemark												x		*	C
Dianthus juniperinus Sm.												x		*	C
Dianthus juniperinus Sm. subsp. *juniperinus*												x		*	C
Dianthus juniperinus subsp. *aciphyllus* (Ser.) Turland												x		*	C
Dianthus juniperinus subsp. *bauhinorum* (Greuter) Turland												x		*	C
Dianthus juniperinus subsp. *heldreichii* Greuter												x		*	C
Dianthus juniperinus subsp. *idaeus* Turland												x		*	C
Dianthus juniperinus subsp. *kavusicus* Turland												x		*	C
Dianthus juniperinus subsp. *pulviniformis* (Greuter) Turland												x		*	C
Dianthus pinifolius Sm.		x	x	x	x	x	x	x	x					BA	H
Dianthus pinifolius subsp. *tenuicaulis* (Turrill) Strid								x						*	H
Dianthus stamatiadae Rech. f.							x							*	C
Dianthus sylvestris subsp. *longicaulis* (Ten.) Greuter & Burdet	x													Me	H
Dianthus sylvestris Wulfen	x	x	x				x							ME	H
Dianthus xylorrhizus Boiss. & Heldr.												x		*	C
Dianthus zonatus Fenzl													x	EM	C
Doronicum columnae Ten.		x			x	x	x	x		x				BC	H
Draba lacaitae Boiss.				x	x									Bk	H
Draba lasiocarpa Rochel	x	x	x	x	x	x	x	x	x					BC	H
Draba parnassica Boiss. & Heldr.					x					x				*	H
Drymocallis halacsyana (Degen) Kurtto & Strid									x					*	H
Dryopteris mindshelkensis Pavlov		x	x	x	x									Me	G
Dryopteris pallida (Bory) Maire & Petitm.	x	x	x	x	x	x	x	x	x	x	x	x	x	Me	G
Dryopteris villarii (Bellardi) Schinz & Thell.		x		x	x		x	x						ME	G
Drypis spinosa L.		x	x	x	x		x			x				Bl	H
Ebenus cretica L.												x		*	C
Edraianthus graminifolius (L.) A. DC.		x	x	x	x		x	x						BI	C
Ephedra foeminea Forssk.	x	x	x	x	x	x	x	x	x	x	x	x	x	Me	P
Epilobium collinum C.C. Gmel.		x	x	x	x		x	x	x	x				Me	H
Eryngium amethystinum L.	x	x	x	x	x	x	x	x		x				Me	H

Eryngium amorginum Rech. f.											x	x		*	H
*Eryngium glomeratu*m Lam.											x	x	x	EM	H
Eryngium ternatum Poir.												x		*	H
Erysimum candicum Snogerup subsp. *candicum*											x	x		*	C
*Erysimum candicu*m subsp. *carpathum* Snogerup												x		*	C
Erysimum corinthium (Boiss.) Wettst.	x			x	x									*	C
Erysimum microstylum Hausskn.	x	x			x	x	x			x				BA	H
Erysimum naxense Snogerup											x			*	H
Erysimum rhodium Snogerup													x	*	C
Erysimum senoneri (Reut.) Wettst. subsp. senoneri										x	x			*	C
Erysimum senoneri subsp. *icaricum* Snogerup													x	*	C
Euphorbia bivonae Steud.				x									x	Me	P
Euphorbia characias L.	x	x	x	x	x	x	x	x	x	x	x	x	x	Me	H
Euphorbia deflexa Sm.		x	x	x	x	x	x	x	x	x		x		Bk	H
Euphorbia dendroides L.	x		x	x	x	x		x		x	x	x	x	Me	P
Euphorbia herniariifolia Willd.		x	x	x	x	x	x			x		x	x	EM	H
Euphorbia kotschyana Fenzl													x	EM	H C
Euphorbia petrophila C.A. Mey.													x	IT	T
Euphorbia sultan-hassei Strid & al.												x		*	P
Ferula communis L.	x	x	x	x	x	x	x	x	x	x	x	x	x	Me	H
Ferula communis subsp. *glauca* (L.) Rouy & Camus	x		x	x	x					x	x	x	x	Me	H
Ferula tingitana L.													x	Me	H
Ferulago thyrsiflora (Sm.) W.D.J. Koch												x		*	H
Festuca olympica J. Vetter							x							*	H
Festuca polita (Halácsy) Tzvelev		x	x	x	x			x		x		x		EM	H
Festuca pseudosupina J. Vetter		x	x	x	x			x		x		x		EM	H
Festuca sipylea (Hack.) Markgr.-Dann.													x	*	H
Festuca spectabilis Bertol.		x	x	x	x	x								Eu	H
Festuca stygia H. Scholz & Strid												x		EM	H
Festuca stygia H. Scholz & Strid				x										*	H
Fibigia clypeata (L.) Medik.		x	x	x	x		x			x				MS	H
Ficus carica L.	x	x	x	x	x	x	x	x	x	x	x	x	x	MS	P
Gagea graeca (L.) Irmisch	x		x	x	x	x				x	x	x	x	BA	G
Galatella cretica Gand.												x	x	EM	H

Galium amorginum Halácsy										x	x		*	H
*Galium anisophyllo*n subsp. *plebeium* (Boiss. & Heldr.) Ehrend.	x	x	x	x		x	x	x					ME	H
Galium anisophyllon Vill.	x	x	x	x		x	x	x					Bk	H
Galium brevifolium Sm.											x	x	EM	T
Galium brevifolium subsp. *insulare* Ehrend. & Schönb.-Tem.											x	x	EM	T
Galium canum DC.											x	x	EM	C
Galium canum subsp. *ovatum* Ehrend.											x	x	EM	C
Galium citraceum Boiss.			x	x					x		x		*	H
Galium cyllenium Boiss. & Heldr.			x										*	H C
Galium degenii Degen	x	x		x	x	x							BA	H
Galium dumosum Boiss.												x	Bk	H
Galium fruticosum Willd.											x		EM	H C
Galium graecum L.											x	x	EM	C
Galium graecum L. subsp. *graecum*											x	x	EM	C
Galium graecum subsp. *pseudocanum* Ehrend.											x	x	*	C
Galium incanum Sm.		x	x	x		x	x	x	x		x	x	EM	H
Galium incurvum Sm.											x		*	H
*Galium kerner*i Degen & Dörfl.						x							Bk	H
Galium monasterium Krendl	x	x											*	H
Galium reiseri Halácsy									x				*	H
Galium thymifolium Boiss. & Heldr.		x	x						x				*	H
Geranium aristatum Freyn & Sint.	x			x		x			x				Bk	G
Geranium macrorrhizum L.	x			x	x	x	x	x	x				ME	G
Geranium subcaulescens DC.	x	x	x	x	x	x			x				Me	G
Geum reptans L.	x												AA	H
Globularia stygia Boiss.			x										*	C
Gymnocarpium robertianum (Hoffm.) Newman			x			x							Ct	G
Gypsophila nana Bory & Chaub.			x	x							x		*	H
Haberlea rhodopensis Friv.							x						Bk	H
Helichrysum amorginum Boiss. & Orph.										x			*	H
Helichrysum heldreichii Boiss.											x		*	C
Helichrysum orientale (L.) Vaill.			x						x	x	x	x	EM	H
Helichrysum sibthorpii Rouy							x						*	H
Helichrysum taenari Rothm.			x										*	H

Heliosperma intonsum (Greuter & Melzh.) Niketić & Stevan.		x												*	H
Heliosperma pusillum (Waldst. & Kit.) Rchb.		x	x	x	x		x							BC	H
Heliosperma pusillum subsp. *albanicum* (K.Malý) Niketić & Stevan.		x	x	x	x		x							Bk	H
Heliosperma pusillum subsp. *chromodontum* (Boiss. & Reut.) Niketić & Stevan.		x					x							*	H
Hellenocarum multiflorum (Sm.) H. Wolff	x	x	x	x	x		x	x	x	x	x	x	x	Me	G
Heptaptera colladonioides Margot & Reut.	x			x	x									*	H
Hesperis balansae E. Fourn.													x	EM	H
Hieracium bifidum Hornem.		x		x	x	x	x	x						ES	H
Hieracium bifidum subsp. *caesiiflorum* (Norrl.) Zahn				x										Eu	H
Hieracium bifidum subsp. *cardiobasis* Zahn							x							Eu	H
Hieracium bifidum subsp. *stenolepis* (Lindeb.) Zahn								x						Eu	H
Hieracium bifidum subsp. *thuringiacum* Zahn								x						BC	H
Hieracium chalcidicum Boiss. & Heldr.		x		x	x		x	x						BA	H
Hieracium chalcidicum Boiss. & Heldr. subsp. *chalcidicum*				x			x	x						Bk	H
Hieracium chalcidicum subsp. *divaricatu*m (Fr.) Greuter				x				x						Bk	H
Hieracium chalcidicum subsp. *macropannosum* (Rech. f. & Zahn) Greuter								x						*	H
Hieracium chalcidicum subsp. *thessalum* (Formánek) Greuter							x							Bk	H
Hieracium dasycraspedum Buttler		x												*	H
Hieracium dimoniei Zahn		x												Bk	H
Hieracium ferdinandi-coburgii J.Wagner & Zahn		x	x				x							*	H
Hieracium gaudryi Boiss. & Orph.		x			x			?						BA	H
Hieracium gaudryi Boiss. & Orph. subsp. *gaudryi*					x									*	H
*Hieracium gaudry*i subsp. *sibthorpianum* Zahn		x												*	H
Hieracium gracilifurcum Zahn							x							*	H
Hieracium graecum Boiss. & Heldr.		x		x	x		x	x	?					*	H
Hieracium graecum Boiss. & Heldr. subsp. *graecum*		x		x	x		x		?					*	H
Hieracium greuteri Gottschl.				x										*	H
Hieracium gymnocephalum Pant.		x												Bk	H
Hieracium heldreichii Boiss.		x		x	x			x	x			x		Bk	H
Hieracium heldreichii Boiss. subsp. *heldreichii*		x		x	x			x						Bk	H
Hieracium heldreichii subsp. *charrelianum* Zahn								x						*	H
Hieracium jankae R. Uechtr.							x	x						BA	H
Hieracium jankae subsp. *macranthelophorum* Rech. f. & Zahn							x	x						*	H
Hieracium jankae subsp. *patentiramum* Rech. f. & Zahn								x						*	H

Hieracium megalothecum Zahn							x				*	H
Hieracium naegelianum Pančić	x	x	x	x		x					BI	H
Hieracium naegelianum Pančić subsp. *naegelianum*	x	x	x	x		x					Bk	H
Hieracium necopinum Buttler	x										*	H
Hieracium pannosum Boiss.	x	x	x	x	x	x	x	x	x		BA	H
Hieracium pannosum Boiss. subsp. *pannosum*	x				x	x	x				BA	H
Hieracium pannosum subsp. *euboeum* (Halácsy) Zahn			x	x					x		*	H
Hieracium pannosum subsp. *friwaldii* (Rchb. f.) Freyn		x		x		x	x	x	x		Bk	H
Hieracium pannosum subsp. *guicciardii* Zahn				x							*	H
Hieracium pannosum subsp. *taygeteum* (Boiss. & Heldr.) Greuter			x	x			x				Bk	H
Hieracium parnassi Fr.	x	x	x	x		x					*	H
Hieracium psaridianum Zahn			x								*	H
Hieracium sartorianum Boiss. & Heldr.	x	x	x	x			x		x		Bk	H
Hieracium sartorianum Boiss. & Heldr. subsp. *sartorianum*	x		x	x			x				Bk	H
Hieracium sartorianum subsp. *koraxense* Zahn				x							*	H
Hieracium scapigerum Boiss. & al.			x	x		x					*	H
Hieracium scardicum Bornm. & Zahn	?					?	x				BA	H
Hieracium schmidtii subsp. *creticum* (Zahn) Greuter										x	*	H
Hieracium schmidtii subsp. *samothracis* (Ade & Schack) Gottschl.								x			*	H
Hieracium schmidtii subsp. *vranjanum* (Zahn) Greuter				x		x					Bk	H
Hieracium schmidtii Tausch				x		x		x		x	EA	H
Hieracium sericophyllum Nejčeff & Zahn	x	x	x	x		x	x	x			BA	H
Hieracium sericophyllum subsp. *buxbaumii* Zahn						x					*	H
Hieracium sericophyllum subsp. *chamaepannosum* Zahn				x							*	H
Hieracium sericophyllum subsp. *eriocomum* Zahn			x			x	x	x			*	H
Hieracium sericophyllum subsp. *fiedleri* Zahn		x	x								*	H
Hieracium sericophyllum subsp. *olenium* Zahn			x	x		x					Bk	H
Hieracium sericophyllum subsp. *pilosius* (Buttler) Greuter			x	x		x	x				*	H
Hieracium sparsum Friv.	x			x		x	x				EA	H
Hieracium sparsum subsp. *acropolianthelum* Rech. f. & Zahn							x				*	H
Hieracium sparsum subsp. *macedonicum* (Boiss. & Orph.) Zahn						x	x				Bk	H
*Hieracium sparsu*m subsp. *naegelianiform*e O. Behr & al.	x					x					Bk	H
Hieracium sparsum subsp. *paniculatissimum* (Zahn) Zahn							x				Bk	H
Hieracium triadanum subsp. *sparsum*						x	x				BA	H

Hieracium triadanum Zahn				x	x			x						*	H
Hieracium trikalense Buttler		x	x											*	H
Hieracium turbinellum Zahn					x			x						*	H
Hieracium waldsteinii subsp. *sublanifolium* Zahn		x												Bk	H
Hieracium waldsteinii subsp. *suborieni* Zahn		x												Bk	H
Hieracium waldsteinii Tausch		x	x				x							Bk	H
Hippocrepis emerus (L.) Lassen	x	x	x	x	x	x	x	x	x	x	x		x	Eu	P
Hippocrepis emerus subsp. *emeroides* (Boiss. & Spruner) Lassen	x	x	x	x	x	x	x	x	x	x	x		x	EM	P
Hornungia petraea (L.) Rchb.	x	x	x	x	x	x	x	x	x	x		x	x	Me	T
Hyoscyamus albus L.	x		x	x	x	x	x	x	x	x	x	x	x	Me	T H
Hyoscyamus aureus L.	x			x							x	x	x	EM	H
Hypericum aciferum (Greuter) N. Robson												x		*	C
Hypericum aegypticum L.	x			x								x		Me	C
Hypericum aegypticum subsp. *webbii* (Spach) N. Robson	x			x								x		Me	C
Hypericum amblycalyx Coustur. & Gand.												x		*	C
Hypericum athoum Boiss. & Orph.								x	x					*	H
Hypericum boehlingraabei Kit Tan & al.				x										*	H
Hypericum cuisinii Barbey												x		*	H
Hypericum empetrifolium subsp. *oliganthum* (Rech. f.) I. Hagemann												x		*	C
Hypericum empetrifolium Willd.	x			x	x	x		x	x	x	x	x	x	EM	C
Hypericum fragile Boiss.					?					x				*	H
Hypericum jovis Greuter												x		*	C
Hypericum montbretii Spach						x	x	x	x					EA	H
Hypericum taygeteum Quézel & Contandr.				x										*	H
Hypochaeris achyrophorus L.	x	x	x	x	x	x	x	x	x	x	x	x	x	Me	T
Hyssopus officinalis L.		x	x				x	x						EA	H
Hyssopus officinalis subsp. *aristatus* (Godr.) Briq.							x	x						EA	H
Iberis runemarkii Greuter & Burdet													x	*	C
Iberis sempervirens L.		x	x	x	x	x	x	x		x		x		Me	C
Inula candida (L.) Cass.				x	x					x		x		*	H
Inula candida (L.) Cass. subsp. *candida*				x								x		*	H
Inula candida subsp. *decalvans* (Halácsy) Tutin												x		*	H
Inula candida subsp. *limonella* (Heldr.) Rech. f.				x	x					x				*	H
Inula subfloccosa Rech. f.										x				*	H

Inula verbascifolia (Willd.) Hausskn.	x		x	x	x	x	x	x	x	x		x	x	BI	H
Inula verbascifolia (Willd.) Hausskn. subsp. *verbascifolia*	x		?											BI	H
Inula verbascifolia subsp. *aschersoniana* (Janka) Tutin	?				?	x	x	x	x					BA	H
Inula verbascifolia subsp. *heterolepis* (Boiss.) Tutin												x	x	EM	H
Inula verbascifolia subsp. *methanaea* (Hausskn.) Tutin				x	x					x				*	H
Inula verbascifolia subsp. *parnassica* (Boiss. & Heldr.) Tutin				x	x		x			x				*	H
Jacobaea maritima (L.) Pelser & Meijden	x		x	x	x			x		x	x	x	x	Me	C
Jacobaea maritima (L.) Pelser & Meijden subsp. *maritima*												x		Me	C
*Jacobaea maritim*a subsp. *bicolor* (Willd.) B. Nord. & Greuter	x		x	x	x			x		x	x		x	Me	C
Jankaea heldreichii (Boiss.) Boiss.							x							*	H
Johrenia dichotoma DC.													x	EM	H
Juniperus communis subsp. *nana* (Willd.) Syme		x	x	x	x	x	x	x		x				Ct	P
Kernera saxatilis (L.) Sweet							x							BC	H
Klasea cretica (Turrill) Holub												x		*	H
Klasea moreana Greuter				x										EM	H
Lactuca acanthifolia (Willd.) Boiss.				x	x					x	x	x	x	EM	C
Lactuca intricata Boiss.		x	x	x	x		x			x			x	EM	H
Lamyropsis carpini Greuter		x												*	G
Leontodon crispus Vill.	x	x	x	x	x	x	x	x	x	x				Me	H
Leontodon hellenicus Phitos			x		x									*	H
Leontodon hispidus L.	x	x	x	x	x	x	x	x	x	x				EA	H
Limonium cornarianum Kypriot. & R. Artelari												x		*	C
Linaria peloponnesiaca Boiss. & Heldr.	x	x	x	x	x	x	x	x		x				Bk	H
Linum arboreum L.											x	x	x	EM	C
Linum caespitosum Sm.												x		*	C
Linum gyaricum subsp. *icaricum* Christod.													x	*	C
Linum gyaricum Vierh. subsp. *gyaricum*										x			x	*	C
Lithodora zahnii (Halácsy) I.M. Johnst.				x										*	P
Lomelosia albocincta (Greuter) Greuter & Burdet												x		*	C
Lomelosia crenata (Cirillo) Greuter & Burdet	x	x	x	x	x		x							Me	C
Lomelosia crenata subsp. *breviscapa* (Boiss. & Heldr.) Greuter & Burdet				x										*	C
Lomelosia crenata subsp. *dallaportae* (Boiss.) Greuter & Burdet	x													BI	C
Lomelosia hymettia (Boiss. & Spruner) Greuter & Burdet				x	x					x				*	C
Lomelosia minoana (P.H. Davis) Greuter & Burdet				?								x		*	C

Lomelosia minoana (P.H. Davis) Greuter & Burdet subsp. *minoana*												x		*	C
Lomelosia minoana subsp. *asterusica* (Greuter) Greuter & Burdet												x		*	C
Lomelosia variifolia (Boiss.) Greuter & Burdet												x	x	*	C
Macrotomia densiflora (Ledeb.) McBride				x	x									MS	C
Malcolmia graeca Boiss. & Spruner	x	x	x	x	x	x	x	x		x				Bk	T
Malcolmia macrocalyx (Halácsy) Rech. f.									x		x			*	T
Marrubium velutinum Sm.		x	x	x	x		x			x				Bk	T
Matthiola incana (L.) R. Br.	x		x	x	x	x		x	x	x	x	x	x	ME	H
Medicago arborea L.	x		x	x	x	x	x	x	x	x	x	x	x	Me	P
Medicago arborea L. subsp. *arborea*	x		x	x	x	x	x	x	x	x	x	x	x	Me	P
Medicago arborea subsp. *strasseri* (Greuter & al.) Sobr.-Vest. & Ceresuela												x		*	P
Medicago truncatula Gaertn.	x	x	x	x	x	x	x	x	x	x	x	x	x	MS	T
Melica ciliata L.	x	x	x	x	x	x	x	x	x	x	x	x	x	MA	H
Melica ciliata L. subsp. *ciliata*			x	x	x	x	x	x		x	x		x	Me	H
Melica ciliata subsp. *glauca* (F. W. Schultz) K. Richt.			x	x	x	x	x	x		x	x		x	Me	H
Melica ciliata subsp. *magnolii* (Gren. & Godr.) K. Richt.				x	x			x	x		x	x	x	MS	H
Melica cretica Boiss. & Heldr.		x	x	x	x		x	x	x	x		x	x	EM	H
Melica cretica Boiss. & Heldr. subsp. *cretica*				x					x			x		EM	H
Melica cretica subsp. *monticola* (Prokudin) W. Hempel		x	x	x	x		x	x				x		EM	H
Melica minuta L.	x		x	x				x	x	x	x	x	x	Me	H
Melica rectiflora Boiss. & Heldr.			x	x							x	x		*	H
Micromeria acropolitana Halácsy					x									*	C
Micromeria carpatha Rech. f.												x		*	C
Micromeria cremnophila Boiss. & Heldr.		x	x	x	x		x							ES	C
Micromeria cristata (Hampe) Griseb.		x					x	x	x					*	C
Micromeria hispida Benth.												x		EM	C
Micromeria myrtifolia Boiss. & Hohen.	x		x	x		x	x	x	x	x	x	x	x	Me	C
Micromeria nervosa (Desf.) Benth.	x		x	x	x	x	x	x	x	x	x	x	x	*	C
Micromeria sphaciotica Benth.												x		*	H
Minuartia juniperina (L.) Maire & Petitm.		x	x	x						x				EM	H
Minuartia pichleri (Boiss.) Maire & Petitm.				x										*	H
Minuartia stellata (E.D. Clarke) Maire & Petitm.			x	x	x					x				Bk	C
Moltkia petraea (Tratt.) Griseb.		x	x		?									Bk	C
Morina persica L.		x	x	x	x	x	x	x		x				MS	H

Myosotis alpestris F.W. Schmidt		x	x	x	x		x	x	x	x				EA	H
Myosotis speluncicola (Boiss.) Rouy		x												Me	T
Nepeta camphorata Boiss. & Heldr.				x										*	H
Nepeta melissifolia Lam.											x	x		*	H
Nepeta parnassica Boiss.			x	x						x				Bk	H
Odontites linkii Boiss.				x	x	x	x			x		x	x	EM	H
Omphalodes luciliae Boiss.				x	x		x	x						EM	H
Omphalodes luciliae subsp. *scopulorum* J.R. Edm.				x	x		x	x						EM	H
Omphalodes runemarkii Strid & Kit Tan				x										*	H
Onobrychis sphaciotica Greuter												x		*	H
Origanum calcaratum Juss.											x	x	x	*	C
Origanum dictamnus L.												x		*	C
Origanum onites L.	x		x	x					x	x	x	x	x	Me	C
Origanum scabrum Boiss. & Heldr.				x						x				*	G
Origanum symes Carlström													x	*	C
Origanum vetteri Briq. & Barbey												x		*	C
Osyris alba L.	x	x	x	x	x	x	x	x	x	x	x	x	x	Me	C
Paragymnopteris marantae (L.) K.H. Shing		x	x	x	x	x	x	x	x	x			x	*	C
Parietaria cretica L.	x	x	x	x	x	x		x	x	x	x	x	x	EM	P
Parietaria judaica L.	x	x	x	x	x	x	x	x	x	x	x	x	x	EA	H
Parietaria lusitanica L.	x	x	x	x	x	x		x	x	x	x	x	x	ME	T
Petromarula pinnata (L.) A. DC.												x		*	H
Petrorhagia dianthoides (Sm.) P.W. Ball & Heywood												x		*	H
Petrorhagia grandiflora Iatroú				x										*	H
Peucedanum arenarium subsp. *neumayeri* (Vis.) Stoj. & Stef.		x					x	x						BA	H
Phagnalon rupestre (L.) DC.	x	x	x	x	x	x		x	x	x	x	x	x	Me	C
Phagnalon rupestre (L.) DC. subsp. *rupestre*	x		x	x	x			x			x	x	x	Me	C
Phagnalon rupestre subsp. *graecum* (Boiss. & Heldr.) Batt.	x	x	x	x	x	x		x	x	x	x	x	x	Me	C
Phagnalon saxatile (L.) Cass.	x			x	x						x	x		Me	C
Phitosia crocifolia (Boiss. & Heldr.) Kamari & Greuter				x										*	H
Phleum alpinum L.		x	x	x	x	x	x	x	x	x				AA	H
Pimpinella pretenderis (Heldr.) Halácsy											x	?		*	H
Plocama calabrica (L. f.) M. Backlund & Thulin	x	x	x	x	x	x	x			x		x	x	Me	C P
Poa cenisia All.		x	x	x	x		x			x		x		Eu	G

Poa thessala Boiss. & Orph.		x	x	x	x	x	x	x	x	x				BA	H
Polygonum icaricum Rech. f.									x				x	*	C
Polypodium cambricum L.	x	x	x	x	x	x	x	x	x	x	x	x	x	MA	G
Polypodium vulgare L.	x	x	x	x	x	x	x	x	x	x				Ct	G
Polystichum lonchitis (L.) Roth		x	x	x	x		x	x		x		x		AA	G
Potentilla arcadiensis Iatroú				x										*	H
Potentilla deorum Boiss. & Heldr.							x							*	H
Potentilla haynaldiana Janka								x						Bk	H
Potentilla kionaea Halácsy					x									*	H
Potentilla speciosa subsp. *illyrica* Soják		x												Bk	H
Potentilla speciosa Willd.		x	x	x	x	x	x	x		x		x		BA	H
Prangos ferulacea (L.) Lindl.		x	x	x	x			?		x				EA	H
Prunus graeca (Lindl.) Steud.													x	EM	P
Prunus prostrata Labill.		x	x	x	x	x	x	x	x	x		x	x	Me	P
Pseudofumaria alba (Mill.) Lidén		x					x							BA	T
Pseudofumaria alba subsp. *leiosperma* (Conrad) Lidén		x					x							Bk	T
Pteris vittata L.	x			x						x		x	x	ST	G
Pterocephalus perennis Coult.	x	x	x	x	x	x	x	x		x				BA	C
Pterocephalus perennis Coult. subsp. *perennis*				x	x	x	x	x		x				*	C
Pterocephalus perennis subsp. *bellidifolius* (Boiss.) Vierh.	x	x	x											Bk	C
Pterocephalus pinardii Boiss.													x	EM	C
Ptilostemon afer (Jacq.) Greuter		x	x	x	x	x	x	x	x	x				BA	H
Ptilostemon chamaepeuce (L.) Less.	x		x	x	x	x	x	x		x	x	x	x	EM	C
Ptilostemon gnaphaloides (Cirillo) Soják	x			x	x			x		x		x	x	Me	C
Ptilostemon gnaphaloides (Cirillo) Soják subsp. *gnaphaloides*	x													Me	C
Ptilostemon gnaphaloides subsp. *pseudofruticosus* (Pamp.) Greuter				x	x			x		x		x	x	EM	C
Ramonda nathaliae Pančić & Petrović							x							Bk	H
Ramonda serbica Pančić		x					x							Bk	H
Ranunculus brevifolius Ten.		x	x	x	x		x			x		x		BI	G
Ranunculus creticus L.											x	x	x	EM	H
Ranunculus incomparabilis Janka							x	x						Bk	H
Ranunculus subhomophyllus (Halácsy) Vierh.				x								x		*	H
Ranunculus thasius Halácsy							x	x	x	x	x		x	*	H
Ranunculus veronicae N. Böhling												x		*	H

Reichardia picroides (L.) Roth	x	x	x	x	x	x	x	x	x	x	x	x	x	Me	H
Rhamnus alpina L.		x	x		x	x	x	x						Eu	P
Rhamnus alpina subsp. *fallax* (Boiss.) Maire & Petitm.		x	x		x	x	x	x						EM	P
Rhamnus lycioides L.	x			x	x	x			x	x	x	x	x	Me	P
Rhamnus lycioides subsp. *graeca* (Boiss. & Reut.) Tutin	x			x	x	x					x	x	x	EM	P
Rhamnus pichleri Bornm.													x	EM	P
Rhamnus pumila Turra					x		x							Me	P
Rhamnus saxatilis Jacq.	x	x	x	x	x	x	x	x	x	x	x	x	x	Eu	P
Rhamnus saxatilis subsp. *prunifolia* (Sm.) Aldén	x	x		x	x		x	x		x	x	x	x	Bk	P
Rhamnus sibthorpiana Schult.				x	x									*	P
Rorippa icarica Rech. f.													x	*	H
Rosa heckeliana Tratt.		x	x	x	x	x	x	x		x		x		BI	C
Rosa pendulina L.		x	x				x	x						Eu	P
Rosa pulverulenta M. Bieb.		x	x	x	x	x	x	x	x	x		x	x	ME	P
Rosmarinus officinalis L.	x		x	x	x	x	x	x	x	x	x	x	x	Me	P
Rosularia serrata (L.) A. Berger				x							x	x	x	EM	H C
Rumex scutatus L.		x	x	x	x		x	x	x	x	x			EA	C
Ruta chalepensis L.	x	x	x	x	x	x	x	x	x	x	x	x	x	Me	C
Ruta chalepensis subsp. *fumariifolia* (Boiss. & Heldr.) Nyman				x							x	x		*	C
Ruta graveolens L.	x	x	x	x	x	x	x	x	x	x	x	x		ME	C
Ruta montana (L.) L.					x								?	Me	C
Samolus valerandi L.	x	x	x	x	x	x	x	x	x	x	x	x	x	Co	H
Sanguisorba cretica Hayek												x		*	H
Sanguisorba rupicola (Boiss. & Reut.) A. Braun & C.D. Bouché							x							Me	H
Satureja athoa K. Malý								x						*	C
Satureja hellenica Halácsy					x					x				*	C
Satureja icarica P.H. Davis													x	*	C
Satureja parnassica Boiss.			x	x	x									EM	C
Satureja parnassica Boiss. subsp. *parnassica*			x	x	x									*	C
Saxifraga adscendens L.	x	x	x	x	x	x	x	x						AA	H
Saxifraga adscendens L. subsp. *adscendens*		x	x		x		x	x						AA	H
Saxifraga adscendens subsp. *parnassica* (Boiss. & Heldr.) Hayek	x	x	x	x	x	x	x	x						BI	H
Saxifraga corymbosa Boiss.		x												BA	H
Saxifraga exarata Vill.		x		x	x		x	x		x				ME	H

Saxifraga federici-augusti Biasol.		x	x				x							BA	H
Saxifraga federici-augusti Biasol. subsp. *federici-augusti*		x												Bk	H
Saxifraga federici-augusti subsp. *grisebachii* (Degen & Dörfl.) D.A. Webb		x					x							Bk	H
Saxifraga ferdinandi-coburgi Kellerer & Sünd.								x						Bk	H
Saxifraga hederacea L.	x		x	x	x	x		x	x	x	x	x	x	EM	T
Saxifraga marginata Sternb.		x	x	x	x									BI	H
Saxifraga oppositifolia L.		x												AA	C
Saxifraga paniculata Mill.		x	x	x	x		x	x		x				AA	H
Saxifraga pedemontana subsp. *cymosa* Engl.							x							BC	C
Saxifraga rotundifolia L.	x	x	x	x	x	x	x	x	x	x		x	x	Eu	H
Saxifraga rotundifolia subsp. *chrysospleniifolia* (Boiss.) D.A. Webb	x	x	x	x	x	x	x	x	x	x		x	x	Bk	H
Saxifraga sancta Griseb.								x						BA	C
Saxifraga scardica Griseb.		x	x	x		x	x			x				Bk	C
Saxifraga sempervivum K. Koch			x	x	x		x	x	x	x				BA	C
Saxifraga sibirica L.									x				x	EA	H
Saxifraga sibthorpii Boiss.				x	x					x				*	H
Saxifraga spruneri Boiss.				x	x		x							BK	C
Saxifraga stribrnyi (Velen.) Podp.								x						Bk	H
Saxifraga taygetea Boiss. & Heldr.		x	x	x	x									BI	H
Saxifraga tridactylites L.	x	x	x	x	x	x	x	x	x	x	x	x	x	EA	T
Scaligeria halophila (Rech. f.) Rech. f.											x	x		*	H
Scaligeria napiformis (Spreng.) Grande	x	x	x	x	x	x	x	x	x	x	x	x	x	EM	H
Scorzonera araneosa Sm.											x			*	H
Scorzonera cretica Willd.		?		x							x	x	x	*	H
Scorzonera scyria M.A. Gust. & Snogerup										x				*	H
Scrophularia canina L.	x	x	x	x	x	x	x	x	x	x	x		x	ME	H
Scrophularia heterophylla Willd.	x		x	x	x	x	x	x	x	x	x	x	x	EM	H
Scrophularia lucida L.	x		x	x	x	x	x	x		x	x	x	x	Me	H
Scrophularia myriophylla Boiss. & Heldr.			x	x										EM	H
Scrophularia peregrina L.	x	x	x	x	x	x	x	x	x	x	x	x	x	Me	T
Scrophularia pinardii Boiss.													x	EM	C
Scutellaria albida L.					x	x	x	x	x		x		x	MA	H
Scutellaria goulimyi Rech. f.										x				*	H
Scutellaria rupestris Boiss. & Heldr.	x	x	x	x	x	x	x			x				Bk	H

Scutellaria rupestris subsp. *adenotricha* (Boiss. & Heldr.) Greuter & Burdet	x	x	x		x		x							Bk	H
Scutellaria rupestris subsp. *caroli-henrici* Bothmer				x										*	H
Scutellaria rupestris subsp. *cephalonica* (Rech. f.) Greuter & Burdet	x													*	H
Scutellaria rupestris subsp. *cytherea* (Rech. f.) Greuter & Burdet				x										*	H
Scutellaria sieberi Benth.												x		*	H
Scutellaria sporadum Bothmer														*	H
Securigera globosa (Lam.) Lassen												x		*	H
Sedum album L.		x	x	x	x	x	x	x	x	x	x	x		EA	C
Sedum annuum L.		x	x		x	x	x	x	x	x				AA	T H
Sedum cepaea L.	x	x	x	x	x	x	x	x	x	x	x	x	x	Me	T
Sedum creticum C. Presl				x								x		*	T C
Sedum dasyphyllum L.		x	x	x	x	x	x	x	x	x				ME	H
Sedum eriocarpum subsp. *apertiflorum* 't Hart	x				x					x				*	H
Sedum eriocarpum subsp. *caricum* (Carlström) 't Hart													x	EM	H
Sedum eriocarpum subsp. *delicum* (Vierh.) 't Hart											x	x	x	*	H
Sedum eriocarpum subsp. *epiroticum* (Bald.) 't Hart	x		x	x	x									*	H
Sedum eriocarpum Sm. subsp. *eriocarpum*				x	x									*	H
Sedum eriocarpum subsp. *orientale* 't Hart													x	EM	H
Sedum eriocarpum subsp. *spathulifolium* 't Hart												x		*	H
Sedum grisebachii Boiss. & Heldr.		x	x				x	x	x					BA	H
Sedum hispanicum L.	x	x	x	x	x	x	x	x	x	x	x	x	x	EA	H C
Sedum laconicum Boiss. & Heldr.	x		x	x	x	x				x		x	?	EM	H C
Sedum laconicum Boiss. & Heldr. subsp. *laconicum*	x		x	x	x	x				x		x	?	*	H
Sedum litoreum Guss.	x		x	x	x	x	x	x	x	x	x	x	x	Me	T
Sedum magellense Ten.		x	x	x	x	x	x	x		x		x		Me	C
Sedum ochroleucum Chaix	x	x	x	x	x	x	x	x	x	x				Me	H
Sedum praesidis Runemark & Greuter												x		*	T
Sedum rubens L.	x	x	x	x	x	x	x	x	x	x	x	x	x		
Sedum stefco Stef.							x	x						Bk	H
Sedum tristriatum Boiss. & Heldr.				x								x		MA	T
Sedum tuberiferum Stoj. & Stef.							x	x						Bk	G
Sedum urvillei DC.	x	x	x	x	x	x	x	x	x	x				ME	H
Selaginella denticulata (L.) Spring	x		x	x	x	x	x	x	x	x	x	x	x	Me	H

Sempervivum ciliosum Craib						x							Bk	C
Sempervivum leucanthum Pančić							x						Bk	C
Sempervivum marmoreum Griseb.		x	x	x	x	x	x						Eu	C
Sempervivum marmoreum Griseb. subsp. *marmoreum*		x	x	x	x	x	x						BC	C
Sempervivum ruthenicum Schnittsp. & C.B. Lehm.						x	x	x					ME	C
Senecio fruticulosus Sm.											x		*	H
Senecio thapsoides DC.			x	x	x		x		x				Bk	H
Seseli aroanicum Hartvig				x									*	H
Seseli crithmifolium (DC.) Boiss.										x	x	x	*	C
Seseli montanum subsp. *tommasinii* (Rchb. f.) Arcang.	?	x				x	?						BI	H
Seseli parnassicum Boiss. & Heldr.			x		x								*	H
Sesleria achtarovii Deyl						x	x	x					Bk	H
Sesleria coerulans Friv.			x	x		x	x						BC	H
Sesleria doerfleri Hayek											x		*	H
Sesleria tenerrima (Fritsch) Hayek		x												
