# Supplementary material for: Linking Taxonomic, Phylogenetic and Functional Plant Diversity with Ecosystem Services of Cliffs and Screes in Greece
Source: Plants (Basel). 2021 May 17;10(5):992. doi: 10.3390/plants10050992 (PMC8156371; doi:10.3390/plants10050992)
Supplement: Supplementary file 1 [file plants-10-00992-s001.zip › Supplementary file Table S2.pdf]

Linking Taxonomic, Phylogenetic and Functional plant diversity with Ecosystem Services of cliffs and screes in Greece

Maria Panitsa*, Ioannis P. Kokkoris, Konstantinos Kougioumoutzis, Anna Kontopanou, Ioannis Bazos, Arne Strid and Panayotis Dimopoulos*

Supplementary file

Table S2. Plant taxa occurring on cliffs and screes in mid- to high-altitudes and their functional traits concerning life form, longevity, growth form, petal color, pollination type and dispersal mode.

Taxon	Longevity	Growth form	Life form	Pollination type	Fruit type	Dispersal mode	Petal Color
Achillea abrotanoides (Vis.) Vis.	perennial	suffruticose	C	entomogamy	achenes	anemochory	white
Achillea ambrosiaca (Boiss. & Heldr.) Boiss.	perennial	caespitose	C	entomogamy	achenes	anemochory	white
Achillea fraasii Sch. Bip.	perennial	caespitose	H	entomogamy	achenes	anemochory	white
Achillea holosericea Sm.	perennial	caespitose	H	entomogamy	achenes	anemochory	yellow
Achillea umbellata Sm.	perennial	subcaespitose	C	entomogamy	achenes	anemochory	white
Acinos alpinus (L.) Moench	perennial	caespitose	H	entomogamy	achenes	barochory	violet with white markings
Aetheorhiza bulbosa (L.) Cass.	perennial	scapose	G	entomogamy	achenes	epizoohory	yellow
Aethionema saxatile (L.) R. Br.	perennial	suffruticose	C	entomogamy	siliques	anemochory	white, pink, purplish or lilac
Alchemilla amphiargyrea Buser	perennial	rosulate	H	apogamy	achenes	zoochory	
Alchemilla aroanica (Buser) Rothm.	perennial	rosulate	H	apogamy	achene	zoochory	yellow
Allium frigidum Boiss. & Heldr.	perennial	scapose	G	entomogamy	membranous capsule	barochory	yellowish or pink
Allium subhirsutum L.	perennial	scapose	G	entomogamy	membranous capsule	barochory	white
Alopecurus gerardii Vill.	perennial	scapose	G	anemogamy	caryopsis	barochory	
Alyssum montanum L.	perennial	caespitose	C	entomogamy	siliques	anemochory	yellow
Amelanchier ovalis Medik.	perennial	fruticose	P	entomogamy	drupe	endozoochory	white
Amelanchier ovalis subsp. *cretica* (Willd.) Maire & Petitm.	perennial	fruticose	P	entomogamy	pome	endozoochore	white
Amelanchier ovalis Medik. subsp. *ovalis*	perennial	fruticose	P	entomogamy	pome	endozoochore	white
Amelanchier parviflora Boiss.	perennial	fruticose	P	entomogamy	berry-like structure	zoochory	
Amelanchier parviflora subsp. *chelmea* (Halácsy) Ziel.	perennial	fruticose	P	entomogamy	berry-like pome	zoochory	

Amelanchier parviflora subsp. *dentata* (Boiss.) K.I. Chr.	perennial	sufruticose	P	entomogamy	small berry	zoochory	
Anthyllis aurea Host	perennial	sufruticose	C	entomogamy	legume	epizoochory	bright yellow
Aquilegia ottonis Boiss.	perennial	caespitose	H	entomogamy	follicles	zoochory	sky-blue to violet blue
Aquilegia ottonis subsp. *amaliae* (Boiss.) Strid	perennial	caespitose	H	entomogamy	follicles	epizoochory	whitish or pale blue
Aquilegia ottonis Boiss. subsp. *ottonis*	perennial	caespitose	H	entomogamy	follicles	epizoochory	whitish or pale blue
Arabis alpina L.	perennial	caespitose	H	entomogamy, autogamy	siliqua	anemochory	white
Arabis bryoides Boiss.	perennial	densely caespitose	H	entomogamy	siliqua	anemochory	white
Arenaria conferta Boiss.	biennial short-lived	caespitose	T	entomogamy	capsule	epizoochory	white
Arenaria cretica Spreng.	perennial	caespitose	C	entomogamy	siliqua	barochory	pale rose-pink
Arenaria filicaulis Fenzl	perennial	caespitose	C H	entomogamy	capsule	barochory	white
Arenaria filicaulis Fenzl subsp. *filicaulis*	perennial	caespitose	C H	entomogamy	capsule	barochory	white
Arenaria filicaulis subsp. *teddii* (Turrill) Strid	perennial	caespitose	C H	entomogamy	capsule	barochory	white
Arenaria fragillima Rech. f.	perennial	densely caespitose	C	entomogamy	capsule	barochory	white (rarely pink or purple)
Arenaria gionae Gustavsson	perennial	caespitose	H	entomogamy	capsule	barochory	white
Arenaria graveolens Schreb.	perennial	caespitose	T	entomogamy	capsule	barochory	white
Asperula aristata L. f.	perennial	caespitose	H	entomogamy	capsule	barochory	yellow
Asperula muscosa Boiss. & Heldr.	perennial	caespitose	H	entomogamy	capsule	barochory	white
Asperula purpurea (L.) Ehrend.	perennial		C	entomogamy	capsule	barochory	red
Asplenium adiantum-nigrum L.	Perennial	rosulate	H	hydrogamy	spores	autochory	
Asplenium aegaeum Lovis & al.	Perennial	rosulate	H	hydrogamy	spores	autochory	
Asplenium ceterach L.	perennial	caespitose	H	hydrogamy	spores	autochory	
Asplenium creticum Lovis & al.	Perennial	rosulate	H	hydrogamy	spores	autochory	
Asplenium cuneifolium Viv.	Perennial	caespitose	H	hydrogamy	spores	autochory	
Asplenium fissum Willd.	Perennial	rosulate	H	hydrogamy	spores	autochory	
Asplenium lepidum C. Presl	Perennial	caespitose	H	hydrogamy	spores	autochory	
Asplenium lepidum subsp. *haussknechtii* (Godet & Reut.) Brownsey	Perennial	rosulate	H	hydrogamy	spores	autochory	
Asplenium lepidum C. Presl subsp. *lepidum*	Perennial	caespitose	H	hydrogamy	spores	autochory	
Asplenium ruta-muraria L.	Perennial	rosulate	H	hydrogamy	spores	autochory	

Asplenium scolopendrium L.	Perennial	rosulate	H	hydrogamy	spores	autochory	
Asplenium scolopendrium subsp. *antri-jovis* (Kümmerle) Brownsey & Jermy	Perennial	rosulate	H	hydrogamy	spores	autochory	
Asplenium septentrionale (L.) Hoffm.	Perennial	caespitose	H	hydrogamy	spores	autochory	
Asplenium trichomanes subsp. *inexpectans* Lovis	Perennial	rhizome caespitose	H	hydrogamy	spores	autochory	
Asplenium trichomanes subsp. *pachyrachis* (H. Christ) Lovis & Reichst.	Perennial	caespitose	H	hydrogamy	spores	autochory	
Asplenium trichomanes subsp. *quadrivalens* D.E. Mey.	Perennial	chizome caespitose	H	hydrogamy	spores	autochory	
Asplenium trichomanes L. subsp. *trichomanes*	Perennial	rhizome caespitose	H	hydrogamy	spores	autochory	
Asplenium viride Huds.	Perennial	rhizome caespitose	H	hydrogamy	spores	autochory	
Astragalus creticus Lam.	perennial	caespitose	C	entomogamy	legume	barochory	pinkish
Astragalus mayeri Micevski	Perennial	fruticose	C	entomogamy	legume	zoochory	
Asyneuma limonifolium (L.) Janch.	perennial	caespitose	H	entomogamy	capsule	barochory	purple
Athamanta densa Boiss. & Orph.	Perennial	caespitose	H	entomogamy	schizocarp	epizoochory	
Aubrieta deltoidea (L.) DC.	perennial	caespitose	C	entomogamy	silique	barochory	purple
Aubrieta erubescens Griseb.	Perennial	caespitose	H	entomogamy	siliqua	barochory	pinkish at first, later white
Aubrieta thessala Boissieu	Perennial	caespitose	H	entomogamy	siliqula	barochory	purple
Aurinia moreana Tzanoud. & Iatroú	Perennial		H	entomogamy	silicula	barochory	yellow
Aurinia saxatilis (L.) Desv.	perennial	caespitose	H	entomogamy	silicula	barochory	yellow
Berberis cretica L.	perennial	caespitose	P	entomogamy	berry	endozoochory	yellow
Bornmuellera baldaccii (Degen) Heywood	perennial	sufruticose	H	entomogamy	silicula		white
Bromus tomentellus Boiss.	perennial	caespitose	H	anemogamy	caryopsis	epizoozhory	
Bupleurum trichopodum Boiss. & Spruner	annual	scapose	T	entomogamy	schizocarp	Barochory	yellow
Campanula hawkinsiana Hausskn. & Heldr.	perennial	caespitose	H	entomogamy	capsule	barochory	blue-violet
Cardamine carnosa Waldst. & Kit.	perennial	caespitose	H	entomogamy	silicues	anemochory	white
Cardamine glauca DC.	annual to perennial	caespitose	T	entomogamy	silicues	anemochory	white
Carduus tmoleus Boiss.	perennial		H	entomogamy, autogamy	achene	anemochory	pink or white
Carum graecum Boiss. & Heldr.	perennial	suffruticose	H	entomogamy	schizocarp	barochory	white or yellowish
*Carum heldreichi*i Boiss.	perennial	scapose	H	entomogamy	schizocarp	barochory	white or yellowish-white

Centranthus longiflorus subsp. *junceus* (Boiss. & Heldr.) I. Richardson	perennial	caespitose	H	entomogamy	achene	anemochory	pink or lilac
Cerastium candidissimum Correns	perennial	caespitose	H	entomogamy	capsule	anemochory	white- to yellowish
Cirsium heldreichii Halácsy	biennial	scapose	H	entomogamy, autogamy	achenes	anemochory	white
Cirsium morinifolium Boiss. & Heldr.	biennial	scapose	H	entomogamy	achenes	anemochory	white
Cotoneaster integerrimus Medik.	perennial	fruticose	P	entomogamy	pome	endozoochory	pink
Cotoneaster nummularius Fisch. & C.A. Mey.	perennial	fruticose	P	entomogamy	pome	zoochory	white
Cotoneaster tomentosus (Aiton) Lindl.	perennial	Fruticose	P	entomogamy	pome	endozoochory	reddish
Crepis neglecta subsp. *graeca* (Vierh.) Rech. f.	annual	scapose	T	entomogamy	achenes	anemochory	yellow
Cyclamen graecum Link	perennial	bulbose	G	entomogamy	capsule	myrmecochory	
Cystopteris alpina (Lam.) Desv.	perennial	caespitose	G	hydrogamy	spores	autochory	
Cystopteris fragilis (L.) Bernh.	perennial	caespitose	G	hydrogamy	spores	autochory	
Daphne oleoides Schreb.	perennial	caespitose	C	entomogamy	drupe	epizoochory	white or cream rarely pink
Dianthus juniperinus Sm.	evergreen perennial	suffruticose	C	entomogamy	capsule	zoochory	pale pink with darker stripes
Dianthus juniperinus subsp. *heldreichii* Greuter	perennial	suffruticose	C	entomogamy	capsule	zoochory	bright pink, with a few purple spots
Dianthus juniperinus subsp. *bauhinorum* (Greuter) Turland	perennial	suffruticose	C	entomogamy	capsule	zoochory	red
Dianthus juniperinus subsp. *pulviniformis* (Greuter) Turland	perennial	suffruticose	C	entomogamy	capsule	zoochory	red
Dianthus pinifolius Sm.	perennial	densely caespitose	H	entomogamy	capsule	zoochory	pinkish-purple to lilac
Dianthus sylvestris Wulfen	perennial	densely caespitose	H	entomogamy	capsule	zoochory	pink or pale pink
Doronicum columnae Ten.	perennial	scapose	H	entomogamy	achenes	anemochory	
Draba lacaitae Boiss.	perennial	densely caespitose	H	entomogamy	siliqua	anemochory	light to vivid yellow
Draba lasiocarpa Rochel	perennial	caespitose	H	entomogamy	siliqua	anemochory	bright yellow
Draba parnassica Boiss. & Heldr.	perennial	densely caespitose	H	entomogamy	satiseptate/like silicles	anemochory	light to vivid yellow
Dryopteris villarii (Bellardi) Schinz & Thell.	perennial	rhizome caespitose	G	hydrogamy	spores	autochory	
Drypis spinosa L.	perennial	caespitose	H		capsule		white or pale pinkish
Ephedra foeminea Forssk.	perennial		P	anemogamy	berry	endozoochory	yellow

Epilobium collinum C.C. Gmel.	perennial	caespitose	H	entomogamy	capsule	anemochory	purplish-pink
Eryngium amethystinum L.	perennial		H	entomogamy, autogamy	schizocarp	epizoozhory	
Erysimum microstylum Hausskn.			H	entomogamy	siliquas	anemochory	yellow
Euphorbia characias L.	perennial	caespitose	H	entomogamy	capsule	anemochory	
Euphorbia deflexa Sm.	perennial	caespitose	H	entomogamy	capsule	anemochory	
Euphorbia herniariifolia Willd.	perennial	caespitose	H	entomogamy	capsule	barochory	
Festuca polita (Halácsy) Tzvelev	perennial	caespitose	H	anemogamy	caryopsis	epizoochory	
Festuca spectabilis Bertol.	perennial	caespitose	H	anemogamy	caryopsis	epizoochory	
Galium incanum Sm.	perennial	caespitose	C	entomogamy	capsule	barochory	white or pink
Galium thymifolium Boiss. & Heldr.	perennial	caespitose	H	entomogamy	capsule	barochory	white
Geranium aristatum Freyn & Sint.	perennial	suffriticose	G	entomogamy	capsule	autochory	pale blue whitish-lilac with lilac veins
Geranium macrorrhizum L.	perennial	scapose	G	entomogamy	capsule	epizoozhory	purplish red
Geranium subcaulescens DC.	perennial	caespitose	G	entomogamy	capsule	autochory	reddish purple
Geum reptans L.	Perennial	caespitose	H	entomogamy	schene-Nutlet	zoochory	yellow
Gypsophila nana Bory & Chaub.	perennial	caespitose	H	entomogamy	sapsule	anemochory	usually very pale pink sometimes persistently white.
Heliosperma pusillum (Waldst. & Kit.) Rchb.	perennial	caespitose	H	entomogamy	sapsule	anemochory	pink or pale pink
Hellenocarum multiflorum (Sm.) H. Wolff	biennial or perennial		G	entomogamy	schizocarp	barochory	white
Hippocrepis emerus (L.) Lassen	perennial	fruticose	P	entomogamy	legume	epizoozhory	yellow
Hornungia petraea (L.) Rchb.	Annual		T		siliqula		white
Hypericum athoum Boiss. & Orph.	perennial	caespitose	H	entomogamy	capsule	barochory	pale yellow
Hypericum empetrifolium Willd.	perennial	caespitose	C	entomogamy	capsule	anemochory	Yellow
Hypericum empetrifolium subsp. *oliganthum* (Rech. f.) I. Hagemann	perennial	caespitose	C	entomogamy	capsule	anemochory	Yellow
Hypochaeris achyrophorus L.	annual	rosulate	T	entomogamy	achenes	anemochory	yellow
Iberis sempervirens L.	perennial	caespitose	C	entomogamy	silique	anemochory	white
Juniperus communis subsp. *nana* (Willd.) Syme	perennial	caespitose	P	anemogamy	berry	endozoochory	

Kernera saxatilis (L.) Sweet	Short-lived perennial.	rosulate	H	entomogamy	siliqula	anemochory	white
Lactuca intricata Boiss.	perennial	caespitose	H	entomogamy	achene	epizoochory	lavender-blue, lilac or white
Leontodon crispus Vill.	perennial	scapose	H	entomogamy	achene	anemochory	yellow
Leontodon hispidus L.	perennial	scapose	H	entomogamy/autogamy	achene	anemochory	yellow
Linaria peloponnesiaca Boiss. & Heldr.	perennial	scapose	H	entomogamy	capsule	anemochory	pale yellow
Linum arboreum L.	perennial	suffruticose	C	entomogamy	capsule	barochory	yellow
Linum caespitosum Sm.	perennial	suffruticose	C	Entomogamy	capsule	barochory	yellow
Malcolmia graeca Boiss. & Spruner	annual	scapose	T	entomogamy	siliques	barochory	pink with yellow base
Malcolmia macrocalyx (Halácsy) Rech. f.	annual	scapose	T	entomogamy	silique	barochory	pink or purple
Marrubium velutinum Sm.		sufruticose	H	entomogamy	achene	epizoozhory	yellow
Medicago truncatula Gaertn.	annual		T	entomogamy	legume	epizoozhory	yellow
Melica ciliata L.	perennial	caespitose	H	anemogamy	caryopsis	anemochory	
Melica minuta L.	perennial	caespitose	H	anemogamy	caryopsis	barochory	
Melica rectiflora Boiss. & Heldr.	perennial	caespitose	H	anemogamy	caryopsis	anemochory	
Minuartia juniperina (L.) Maire & Petitm.	perennial	caespitose	H	entomogamy	capsule	barochory	white
Minuartia stellata (E.D. Clarke) Maire & Petitm.	perennial	caespitose	C	entomogamy	capsule	epizoozhory	white
Morina persica L.	perennial	scapose	H				
Myosotis alpestris F.W. Schmidt	perennial	scapose	H	entomogamy	achenes	epizoozhory	blue
Nepeta parnassica Boiss.	perennial		H	entomogamy	achenes	barochory	purple-blue
Onobrychis sphaciotica Greuter	Perennial	caespitose	H	Entomogamy	legume	zoochory	pinkish-purple
Origanum onites L.	perennial	sufruticose	C	entomogamy	achenes	barochory	white
Osyris alba L.	perennial		P	entomogamy	drupe	endozoochory	yellowish
Parietaria cretica L.	annual	scapose	T,H	anemogamy	achene	epizoozhory	
Peucedanum arenarium subsp. *neumayeri* (Vis.) Stoj. & Stef.	Perennial	Scapose	H	Entomogamy	schizocarp	anemochory	white to coral-pink
Phleum alpinum L.	perennial	caespitose	H	anemogamy	caryopsis	epizoozhory	
Poa cenisia All.	perennial	caespitose	G	anemogamy	caryopsis	barochory	green
Poa thessala Boiss. & Orph.	perennial	caespitose	H	anemogamy	caryopsis	barochory	green
Polygonum icaricum Rech. f.	Perennial	sufruticose	C	Autogamy	achenes	barochory	pink or white
Polypodium vulgare L.	Perennial	Rosulate	G	Hydrogamy	Spores	autochory	
Polystichum lonchitis (L.) Roth	perennial	caespitose	H	Hydrogamy	spores	autochory	

Potentilla deorum Boiss. & Heldr.	Perennial	caespitose	H	Entomogamy	achenes	barochory	white or suffused with pink
Potentilla haynaldiana Janka	Perennial	caespitose	H	Entomogamy	achenes	barochory	white
Potentilla kionaea Halácsy	Perennial	caespitose	H	Entomogamy	achene	barochory	purple
Potentilla speciosa Willd.	perennial	caespitose	H	entomogamy	achenes	barochory	yellow
*Prangos ferulace*a (L.) Lindl.	perennial		H	Entomogamy	schizocarp	barochory	yellow
Prunus prostrata Labill.	perennial	suffruticose	P	entomogamy	drupe	endozoochory	pink
Ptilostemon afer (Jacq.) Greuter	biennial		H	entomogamy	achene	barochory	pink
Ranunculus brevifolius Ten.	perennial	suffruticose	G	entomogamy	achene	anemochory/epizoochory	yellow
Ranunculus incomparabilis Janka	Perennial	caespitose	H	Entomogamy	achenes	barochory	yellow
*Ranunculus subhomophyllu*s (Halácsy) Vierh.	Perennial	caespitose	H	Entomogamy	achene	barochory	Yellow
Rhamnus alpina L.	Perennial	suffruticose	P	anemogamy	fruit with (2-)3(-4) dehiscent pyrenes	endozoochory	
Rhamnus saxatilis Jacq.	perennial	suffruticose	P	anemogamy	drupe	endozoochory	yellow
Rosa heckeliana Tratt.	perennial	suffruticose	C	entomogamy	achene		pink
Rosa pendulina L.	Perennial	fruticose	P	Entomogamy	achene/berry-like structure	endozoochory	deep purplish-pink
Rosa pulverulenta M. Bieb.	perennial	suffruticose	P	entomogamy	achene		pink or white
Rumex scutatus L.	perennial	suffruticose	C	anemogamy	achene	anemochory	white-green
Saxifraga adscendens L.	Biennial	caespitose	H	Entomogamy	capsule	barochory	white
Saxifraga adscendens L. subsp. *adscendens*	Biennial	caespitose	H	Entomogamy	capsule	barochory	white
Saxifraga adscendens subsp. *parnassica* (Boiss. & Heldr.) Hayek	Biennial	caespitose	H	Entomogamy	capsule	barochory	white
Saxifraga corymbosa Boiss.	Perennial	caespitose	H	Entomogamy	capsule	barochory	greenish-yellow and yellow
Saxifraga exarata Vill.	perennial	caespitose	H	Entomogamy	capsule	barochory	yellowish-green to pure white
Saxifraga marginata Sternb.	Perennial	caespitose	H	Entomogamy	capsule	barochory	white or pale pink
Saxifraga oppositifolia L.	Perennial	caespitose	C	Entomogamy	capsule	zoochory	reddish-purple
Saxifraga paniculata Mill.	perennial	densely caespitose	H	entomogamy	capsule	barochory	white or pale cream, with or without small reddish dots.
Saxifraga pedemontana subsp. *cymosa* Engl.	Perennial	caespitose	C	Entomogamy	capsule	barochory	white
Saxifraga rotundifolia L.	Perennial	scapose	H	entomogamy	capsule	barochory	white with reddish-purple

							and sometimes yellow dots above
Saxifraga rotundifolia subsp. *chrysospleniifolia* (Boiss.) D.A. Webb	Biennial	scapose	H	entomogamy	capsule	barochory	Petals with faint yellowish and purple dots, or sometimes lacking dots.
Saxifraga sancta Griseb.	Perennial	caespitose	C	Entomogamy	capsule		yellow
Saxifraga scardica Griseb.	evergreen perennial	rosulate	C	Entomogamy	capsule	barochory	white or sometimes tinged with pink, often aging to a deep cerise-pink.
Saxifraga sempervivum K. Koch	perennial	rosulate	C	Entomogamy	capsule	barochory	reddish-purple
Saxifraga sibirica L.	Perennial	caespitose	H	Entomogamy	capsule	barochory	white
Saxifraga sibthorpii Boiss.	Annual to short-lived perennial	caespitose	H	Entomogamy	capsule	barochory	bright yellow
Saxifraga spruneri Boiss.	Perennial	caespitose	C	Entomogamy	capsule	barochory	white
Saxifraga stribrnyi (Velen.) Podp.	Perennial	rosulate	H	Entomogamy	capsule		purplish-pink
Saxifraga taygetea Boiss. & Heldr.	Perennial	caespitose	H	Entomogamy	capsule	barochory	white spotted with red towards their bases.
Saxifraga tridactylites L.	Annual	ccapose	T	entomogamy	capsule	anemochory	
Scaligeria napiformis (Spreng.) Grande	biennial	scapose	H		schizocarp		white
Scrophularia canina L.	perennial	suffruticose	H	Entomogamy	capsule	barochory	dark purplish-red
Scrophularia heterophylla Willd.	perennial	suffruticose	H	Entomogamy	capsule	barochory	reddish-purple to greenish
Scrophularia lucida L.	biennial	suffruticose	H	Entomogamy	capsule	barochory	greenish-brown
Scrophularia myriophylla Boiss. & Heldr.	biennial	scapose	H	entomogamy	capsule	barochory	greenish-brown
Scutellaria albida L.	perennial	scapose	H	Entomogamy	achene	barochory	white
Scutellaria rupestris Boiss. & Heldr.	perennial		H	Entomogamy	achene	barochory	
Scutellaria sporadum Bothmer	perennial		H	Entomogamy	achene	barochory	

Sedum album L.	perennial	succulente	C	entomogamy, autogamy	follicles	anemochory	white or pink
Sedum annuum L.	Annual or biennial	succulente	T H	Entomogamy	follicles	anemochory	yellow
Sedum cepaea L.	Annual or Biennial sometimes perennial	succulente	T	entomogamy	follicles	anemochory	white
Sedum creticum C. Presl	Annual or biennial	succulente	T C	Entomogamy, autogamy	follicles	Anemochory	greenish-white
Sedum dasyphyllum L.	Perennial	succulente	H	Entomogamy	follicles	anemochory	white or pale pink
Sedum eriocarpum Sm. subsp. *eriocarpum*	Annual	succulente	H	Entomogamy	follicles	anemochory	white
Sedum hispanicum L.	Annual sometimes biennial or perennial	succulente	H C	entomogamy	follicles	anemochory	white
Sedum laconicum Boiss. & Heldr.	Perennial	succulente	H C	Entomogamy	follicles	anemochory	yellow
Sedum laconicum Boiss. & Heldr. subsp. *laconicum*	Perennial	succulente	H	Entomogamy	follicles	anemochory	yellow
Sedum litoreum Guss.	annual	succulente	T	entomogamy	follicles	anemochory	yellow
Sedum magellense Ten.	perennial	succulente	C	entomogamy	follicles	anemochory	whitish
Sedum ochroleucum Chaix	Perennial	succulente	H	Entomogamy	follicles	anemochory	yellow
Sedum rubens L.	annual	succulente	T	Entomogamy	follicles	anemochory	white or pink
Sedum stefco Stef.	Perennial	succulente	H	Entomogamy, autogamy	follicles	anemochory	white or pink
Sedum tristriatum Boiss. & Heldr.	Perennial	succulente	C	Entomogamy, autogamy	follicles erect	anemochory	white
Sedum tuberiferum Stoj. & Stef.	Perennial	succulente	G	entomogamy, autogamy	follicles	anemochory	yellow
Sedum urvillei DC.	perennial	succulente	H	Entomogamy	follicles	anemochory	yellow
Sempervivum ciliosum Craib	perennial	succulente	C	Entomogamy	follicles	barochory	yellow
Sempervivum marmoreum Griseb.	Perennial	succulente	C	Entomogamy	follicles	barochory	red with white margins
Sempervivum ruthenicum Schnittsp. & C.B. Lehm.	Perennial	succulente	C	Entomogamy	follicles	barochory	bright yellow to greenish yellow, purple at the base
Senecio fruticulosus Sm.	perennial	suffruticose	H	entomogamy/autogamy	achenes	anemochory	yellow

Senecio thapsoides DC.	perennial	suffruticose	H	entomogamy/autogamy	achenes	anemochory	yellow
Seseli aroanicum Hartvig	perennial	Scapose	H	Entomogamy	schizocarp	barochory	white to pinkish
Seseli parnassicum Boiss. & Heldr.	perennial	caespitose	H	entomogamy	schizocarp	barochory	white
Sesleria coerulans Friv.	perennial	caespitose	H	anemogamy	caryopsis	barochory	
Sesleria tenerrima (Fritsch) Hayek	perennial	caespitose	H	anemogamy	caryopsis	barochory	
Sesleria vaginalis Boiss. & Orph.	perennial	caespitose	H	anemogamy	caryopsis	barochory	
Sideritis clandestina (Bory & Chaub.) Hayek	perennial	caespitose	H	entomogamy	achene	epizoozhory	yellow
Sideritis raeseri Boiss. & Heldr.	perennial	caespitose	H	entomogamy	achene	epizoozhory	yellow
Silene auriculata Sm.	Perennial	caespitose	H	Entomogamy	capsule	anemochory	white or cream
Silene barbeyana Boiss.	Perennial	densely caespitose	T	Entomogamy	capsule	anemochory	white or pale pink above, pinkish-purple beneath
Silene caesia Sm.	perennial	suffruticose	H	anemogamy	capsule	anemochory	white or pink
Silene dirphya Greuter & Burdet	Perennial	caespitose	H	Entomogamy	capsule	anemochory	white or pinkish
Silene fabarioides Hausskn.	biennial	scapose	H	entomogamy	capsule	anemochory	white
Silene flavescens subsp. *thessalonica* (Boiss. & Heldr.) Nyman	Perennail	caespitose	C	Entomogamy	capsule	anemochory	yellow
Silene haussknechtii Hausskn.	annual	scapose	T	entomogamy	capsule	anemochory	pink
Silene integripetala Bory & Chaub. subsp. *integripetala*	Annual	scapose	T	Entomogamy	capsule	anemochory	bright pink
Silene multicaulis Guss.	perennial	Densely Caespitose	H	Entomogamy	capsule	anemochory	whitish above, greenish to reddish-brown beneath;
Silene multicaulis Guss. subsp. *multicaulis*	perennial	Densely caespitose	H	Entomogamy	capsule	anemochory	whitish above, greenish or reddish-brown beneath
Silene orphanidis Boiss.	Perennial	Caespitose	H	Entomogamy	capsule	anemochory	white above, purplish beneath
Silene parnassica Boiss. & Spruner	perennial	Densely Caespitose	H	entomogamy	capsule	anemochory	white
Silene taygetea Vierh.	Perennial	Caespitose	H	Entomogamy	capsule	anemochory	
Silene vulgaris (Moench) Garcke	Perennial	Caespitose	H C	entomogamy	capsule	anemochory	whitish

Silene waldsteinii Griseb.	perennial	Densely Caespitose	H C	Entomogamy	capsule	anemochory	white
Sorbus aria (L.) Crantz	Perennial	Suffruticose	P	Entomogamy	pome	endozoochory	white
Sorbus umbellata (Desf.) Fritsch	Perennial	Fruticose	P	Entomogamy	Pomes	endozoochory	white
Thamnosciadium junceum (Sm.) Hartvig	perennial	caespitose	G	entomogamy	schizocarp	barochory	white
Theligonum cynocrambe L.	annual	scapose	T	entomogamy	achene	barochory	green
Thymus boissieri Halácsy	perennial	caespitose	C	entomogamy	achene	epizoozhory	pink
Thymus praecox Opiz	perennial	caespitose	C	entomogamy	achene	epizoozhory	whitish to rose-purple
Thymus hartvigii R. Morales	perennial	caespitose	C	entomogamy	achene	epizoozhory	pink
Thymus teucrioides Boiss. & Spruner	perennial	caespitose	C	entomogamy	achene	epizoozhory	purple
Trinia glauca (L.) Dumort.	perennial		H	entomogamy	schizocarp	barochory	whitish
Valantia aprica (Sm.) Tausch	perennial	caespitose	H	entomogamy	capsule	epizoochory	yellowish-white to pinkish
Valantia hispida L.	annual	scapose	T	entomogamy	capsule	epizoozhory	white
Valantia muralis L.	annual	scapose	T	entomogamy	capsule	epizoozhory	yellowish green
Verbascum longifolium Ten.	Biennial or perennial		H	entomogamy	capsule	epizoozhory	yellow
Verbascum spinosum L.	perennial	caespitose	C	entomogamy	capsule	epizoozhory	yellow
Veronica cymbalaria Bodard	annual	scapose	T	entomogamy/autogamy	capsule	epizoozhory	white
Viola albanica Halácsy	perennial	caespitose	H	entomogamy	capsule	myrmecochory	violet
Viola athois W. Becker	Perennial	Caespitose	H	Entomogamy	capsule	myrmecochory	violet
Viola cephalonica Bornm.	Perennial	Caespitose	H	Entomogamy	capsule	myrmecochory	light bluish-violet -pale violet
Viola chelmea Boiss. & Heldr.	Perennial	Caespitose	H	Entomogamy	capsule	myrmecochory	Pale violet
Viola delphinantha Boiss.	Perennial	Caespitose	C	Entomogamy	capsule	myrmecochory	reddish-lilac or pinkish
Viola heldreichiana Boiss.	Annual	Scapose	T	Entomogamy	capsule	myrmecochory	pale violet
Viola mercurii Halácsy	annual	Caespitose	T	Entomogamy	capsule	myrmecochory	yellow
Viola perinensis W. Becker	Perennial	Caespitose	H	Entomogamy	capsule	myrmecochory	yellow or violet with a yellow centre
Viola poetica Boiss. & Spruner	Perennial	Caespitose	H	Entomogamy	capsule	myrmecochory	
Viola pyrenaica DC.	perennial	Rosulate	H	Entomogamy	capsule	myrmecochory	dark violet
