# Supplementary material for: Linking Taxonomic, Phylogenetic and Functional Plant Diversity with Ecosystem Services of Cliffs and Screes in Greece
Source: Plants (Basel). 2021 May 17;10(5):992. doi: 10.3390/plants10050992 (PMC8156371; doi:10.3390/plants10050992)
Supplement: Supplementary file 1 [file plants-10-00992-s001.zip › Supplementary file_Figure S2.pdf]

Linking Taxonomic, Phylogenetic and Functional plant diversity with Ecosystem Services of cliffs and screes in Greece

Maria Panitsa*, Ioannis P. Kokkoris, Konstantinos Kougioumoutzis, Anna Kontopanou, Ioannis Bazos, Arne Strid and Panayotis Dimopoulos,*

Supplementary file

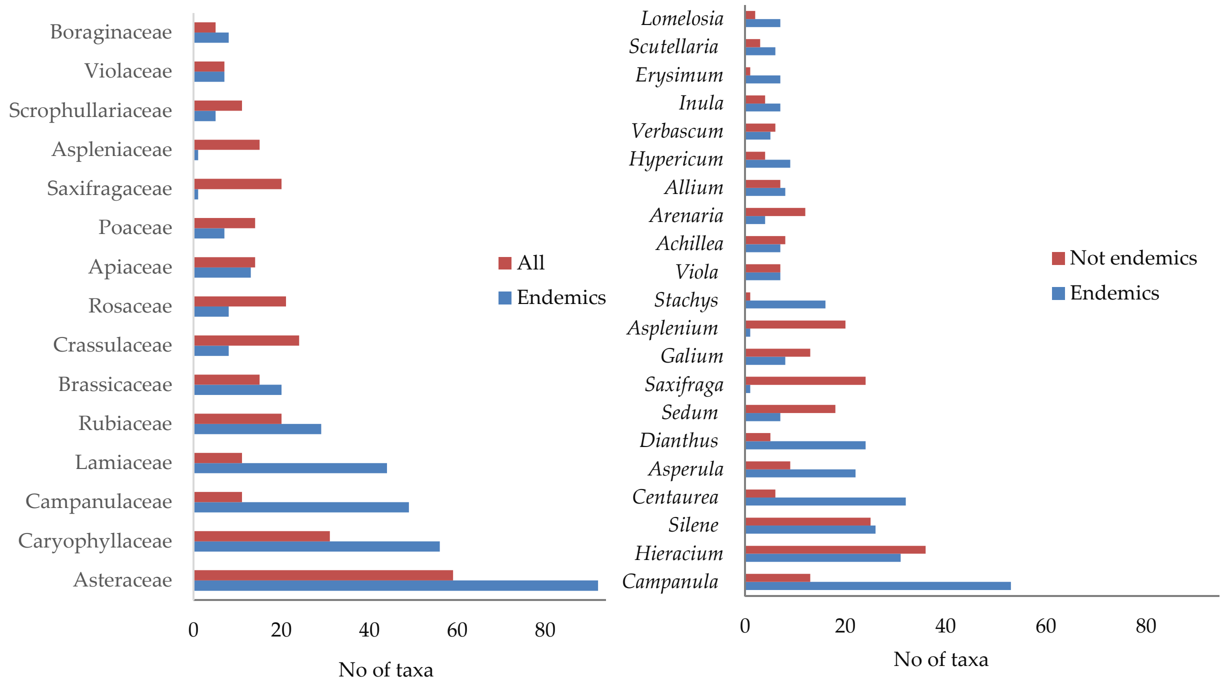

(a) (b)

Figure S2. a. The most taxon-rich families of the floristic composition of cliffs and screes. **b.** The most taxon-rich genera of the floristic composition of cliffs and screes. All = total flora of cliffs and screes ((exclusive and non-exclusive).
