# Supplementary material for: Linking Taxonomic, Phylogenetic and Functional Plant Diversity with Ecosystem Services of Cliffs and Screes in Greece
Source: Plants (Basel). 2021 May 17;10(5):992. doi: 10.3390/plants10050992 (PMC8156371; doi:10.3390/plants10050992)
Supplement: Supplementary file 1 [file plants-10-00992-s001.zip › Supplementary file_Figure S4.pdf]

Linking Taxonomic, Phylogenetic and Functional plant diversity with Ecosystem Services of cliffs and screes in Greece

Maria Panitsa*, Ioannis P. Kokkoris, Konstantinos Kougioumoutzis, Anna Kontopanou, Ioannis Bazos, Arne Strid and Panayotis Dimopoulos,*

Supplementary file

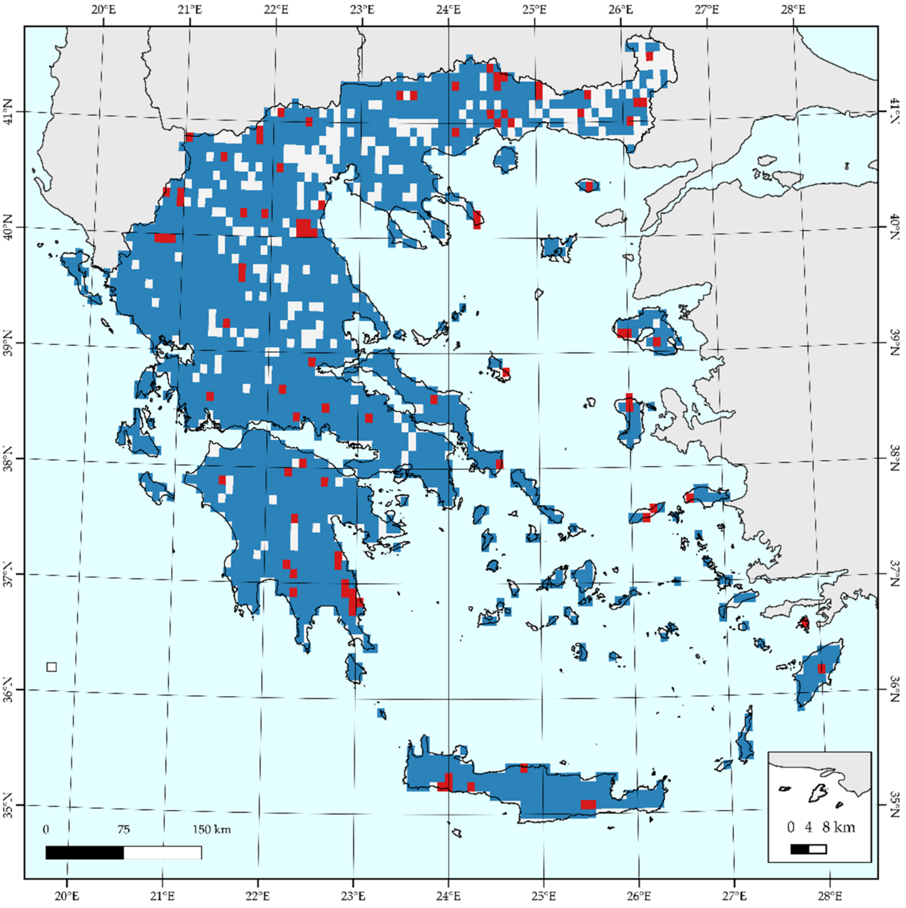

Figure S4. Randomization results (999 runs) for the CWE metric. Red coloring indicates areas with statistically significantly higher than expected corrected weighted endemic richness. Blue coloring indicates areas where the CWE values are not statistically significantly higher or lower than expected.
