# Supplementary material for: Linking Taxonomic, Phylogenetic and Functional Plant Diversity with Ecosystem Services of Cliffs and Screes in Greece
Source: Plants (Basel). 2021 May 17;10(5):992. doi: 10.3390/plants10050992 (PMC8156371; doi:10.3390/plants10050992)
Supplement: Supplementary file 1 [file plants-10-00992-s001.zip › Supplementary file_Figure S5.pdf]

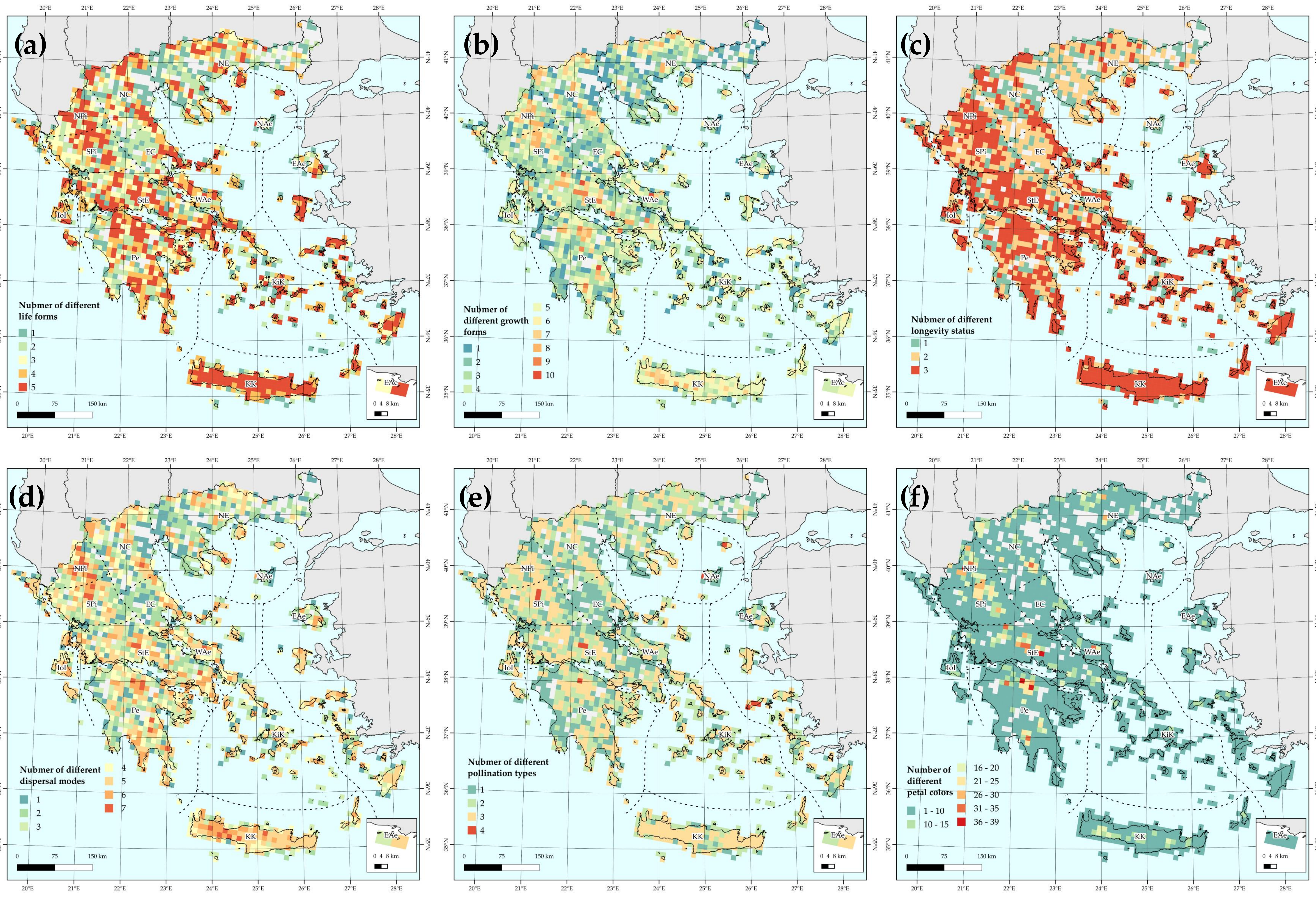

Figure S5. Functional diversity patterns for: life form (a), growth form (b), longevity (c), dispersal mode (d), pollination 260 type (e) and petal color (f), per 10 × 10 km EEA reference grid cell.
